# Williams-Beuren Syndrome Related Methyltransferase WBSCR27: From Structure to Possible Function

**DOI:** 10.3389/fmolb.2022.865743

**Published:** 2022-06-15

**Authors:** Sofia S. Mariasina, Chi-Fon Chang, Tsimafei L. Navalayeu, Anastasia A. Chugunova, Sergey V. Efimov, Viktor G. Zgoda, Vasily A. Ivlev, Olga A. Dontsova, Petr V. Sergiev, Vladimir I. Polshakov

**Affiliations:** ^1^ Faculty of Fundamental Medicine, M.V. Lomonosov Moscow State University, Moscow, Russia; ^2^ Institute of Functional Genomics, M.V. Lomonosov Moscow State University, Moscow, Russia; ^3^ Genomics Research Center, Academia Sinica, Taipei, Taiwan; ^4^ Chemical Department, M.V. Lomonosov Moscow State University, Moscow, Russia; ^5^ NMR Laboratory, Institute of Physics, Kazan Federal University, Kazan, Russia; ^6^ Institute of Biomedical Chemistry, Moscow, Russia; ^7^ Pharmacy Resource Center, RUDN University, Moscow, Russia; ^8^ Skolkovo Institute of Science and Technology, Moscow, Russia

**Keywords:** Williams-Beuren syndrome (WBS), methyltransferase (MTase), NMR, protein structure in solution, protein dynamics, S-adenosyl-L-homocysteine (SAH)

## Abstract

Williams-Beuren syndrome (WBS) is a genetic disorder associated with the hemizygous deletion of several genes in chromosome 7, encoding 26 proteins. Malfunction of these proteins induce multisystemic failure in an organism. While biological functions of most proteins are more or less established, the one of methyltransferase WBSCR27 remains elusive. To find the substrate of methylation catalyzed by WBSCR27 we constructed mouse cell lines with a *Wbscr27* gene knockout and studied the obtained cells using several molecular biology and mass spectrometry techniques. We attempted to pinpoint the methylation target among the RNAs and proteins, but in all cases neither a direct substrate has been identified nor the protein partners have been detected. To reveal the nature of the putative methylation substrate we determined the solution structure and studied the conformational dynamic properties of WBSCR27 in apo state and in complex with S-adenosyl-L-homocysteine (SAH). The protein core was found to form a canonical Rossman fold common for Class I methyltransferases. N-terminus of the protein and the β6–β7 loop were disordered in apo-form, but binding of SAH induced the transition of these fragments to a well-formed substrate binding site. Analyzing the structure of this binding site allows us to suggest potential substrates of WBSCR27 methylation to be probed in further research.

## Introduction

Williams-Beuren syndrome (WBS) is a complex developmental disorder, induced by haploinsufficiency of 24–26 genes in the chromosome region 7q11 (Schubert, 2009). Multisystem disorders associated with this disease include aortic stenosis, hypercalcemia, impaired glucose metabolism, thyroid dysfunction, growth retardation, characteristic facial appearance, mental deficiency, and “friendly” personality which is usually considered as hyper-friendliness (Jones et al., 2000; Pober, 2010; Masserini et al., 2013).

For some phenotypic features, the impact of a specific gene deletion is already well-established. For instance, the ELN gene, being a part of WBS deletion, encodes the protein elastin, a component of vascular walls, and its insufficiency leads to aortic stenosis (Ewart et al., 1993). Deletion of gene LIMK1, encoding LIM-kinase 1, brings about impaired visuospatial constructive cognition (Frangiskakis et al., 1996). There is also solid evidence on the insufficiency of BAZ1B contributing to hypercalcemia through interaction with the vitamin D receptor (Kitagawa et al., 2003). However, the impact of other genes lost in case of WBS manifestation remains unclear.

Uncovering the physiological consequences of gene loss on the behavior characteristic phenotype of WBS patients is among the most complicated directions in WBS studies. There is no direct evidence, but the decreased expression level of some gene products from the WBS chromosome region is likely to be involved. Interestingly, domestic dogs exhibit some of the behavioral traits typical of humans with WBS (vonHoldt et al., 2017): as compared to their ancestor, the gray wolf, domestic dogs have heightened propensity to initiate social contacts showing “hyper-sociability.” Comparing the dog genome to the Yellowstone gray wolf one revealed mobile element insertions affecting transcriptional regulation in the genes responsible for WBS (vonHoldt et al., 2018). Transcriptome sequencing confirmed that the expression levels of six genes placed in WBS chromosome region (WBSCR17, LIMK1, GTF2I, WBSCR27, BAZ1B, and BCL7B) differ between these animals accounting for different behavior patterns.

One of the plausible candidate proteins associated with the behavioral aspects of WBS is WBSCR27. Human and chimpanzee genome sequences were compared and nine human-specific frameshift mutations were identified (Hahn and Lee, 2005). One of these mutations is placed within the WBSCR27 gene coding sequence: there is an 11 bp insertion in human WBSCR27. The insertion occurred specifically in the human lineage and probably could somehow affect the functioning of the protein; and thereby directly or indirectly alter human social behavior in comparison with chimpanzees. However, there is no direct experimental proof of this hypothesis yet.

There is a dearth of information about the biological function of WBSCR27. To outline some functional role of this protein only differential gene expression was measured. The expression level of WBSCR27 was reported to change in response to different external conditions. The overexpression of WBSCR27 was found in three tumor types: esophageal carcinoma, stomach adenocarcinoma, and kidney renal papillary cell carcinoma (Campeanu et al., 2021) by bioinformatic analysis of data available in TCGA (The Cancer Genome Atlas). WBSCR27 is overexpressed also in colon cancer and can be used as a prognostic marker of this desease (Wang et al., 2022).

Salvianolic acid B treatment was recently studied as a potential therapeutic approach for obesity (An et al., 2019). To examine the differential gene expression in mouse white adipose tissue caused by treating with Salvianolic acid B RNA-Seq was performed demonstrating that 234 lncRNAs, 19 circRNAs, and 132 mRNAs were differentially expressed. Among the mRNAs, the upregulated expression of WBSCR27 was the highest, with a fold change of 2.053. These results were confirmed by the qPCR. The other upregulated protein-coding genes were involved in the insulin resistance pathway, while the downregulated genes mainly participated in the IL-17 signaling pathway.

Nevertheless, these data do not shed any light on the possible role of WBSCR27 in WBS, as well as on its biological function in general. Metzger and colleagues (Metzger et al., 2019) previously identified C21orf127 MTase (later renamed to KMT9) to be responsible for histone lysine methylation. Surprisingly, this protein turned out to be a seven-β-stranded methyltransferase (MTase), while all histone lysine methylating proteins known before belonged to SET-domain family (Husmann and Gozani, 2019). To find out other histone lysine MTases within the seven-β-stranded MTase family, cluster analysis on multiple amino acid sequence alignments of putative seven-β-stranded MTase domains was performed. WBSCR27 was found among the seven closest homologs of C21orf127 and was tested for histone methylation in the *in vitro* assay, but did not show any methylation activity.

In our previous work (Mariasina et al., 2020) we demonstrated that WBSCR27 effectively interacts with the cofactor S-(5′-adenosyl)-L-methionine (SAM) and has a canonical Rossman fold, typical of Class I MTases. This information supports the bioinformatic assignment of WBSCR27 to MTases; however, the substrate of methylation catalyzed by this enzyme is still unknown. Here we determined the solution structure of WBSCR27 in apo-state and in complex with the cofactor. This work may shed light on the possible biological role of this protein making the complete mapping of gene deletions in WBS and physiological consequences one step closer.

## Materials and Methods

### WBSCR27 Expression and Purification

The uniformly ^15^N and ^15^N/^13^C enriched protein was expressed in *E. coli* cells grown on ^15^N or ^15^N/^13^C M9 minimal media using the glucose-^13^C (2 g/L) and/or ammonium sulphate-^15^N (1 g/L) as a source of stable isotopes. The protein selectively ^13^C-labelled in the methyl groups of Thr and Met residues was expressed in ^15^N M9 media containing 100% D_2_O and ISOGRO®-D supplemented by Met-ε-^13^СH_3_ and Thr-γ-^13^СH_3_ as well as fully deuterated 2-ketobutyrate and Gly-d_2_ to prevent cross-labelling (Kerfah et al., 2015).

Protein samples were purified as described in Mariasina et al. (2018). Refolding was an important step in the purification procedure of the apo-form of the protein. In this case after affinity chromatography on the Ni-NTA column and subsequent His-tag cleavage, the protein samples were denatured in 6 M urea, washed from endogenous SAH by dialysis, and refolded back to the native form (Mariasina et al., 2018). Samples of the WBSCR27-SAH complex with identical content of the ^13^C and ^15^N isotopes in both protein and ligand were purified without refolding. In all other cases samples for NMR structural studies were prepared by adding the corresponding ligand to the apo-form of WBSCR27 followed by the dialysis against the buffer containing 50 mM NaCl, 50 mM sodium phosphate (pH 7.0), 10 mM DTT and 0.02% NaN_3_.

### Synthesis of [Methyl ^13^C]-SAM and Preparation of ^13^C,^15^N Uniformly Labelled SAH

[Methyl ^13^C]-labelled SAM was synthesized from SAH (Sigma) and ^13^CH_3_I (Cambridge Isotope Laboratories) according to the described method (Huber et al., 2016). 15 mg of SAH were dissolved in 500 µl of deuterated formic acid. 300 µl of ^13^CH_3_I were added to the resulting solution. The mixture was vortexed for 2 h and then stirred at room temperature in the dark. The completeness of the reaction was monitored by ^1^H NMR. After 5 days, 1 ml of water was added and the unreacted ^13^C methyl iodide was extracted with 2 ml of diethyl ether twice. The pH of the aqueous phase was adjusted to 7.15, after which the sample was applied to an ion exchange chromatographic column (732-0003 BioRad cartridge) preliminarily equilibrated with 0.01 M sodium phosphate buffer solution (pH 7.15). The column was washed with 55 ml of the same buffer solution (at first, the uncharged unreacted SAH and subproduct MTA were washed off, then the positively charged SAM was washed off). Next, the column was washed first with 20 ml of 0.1 M acetic acid, then with 20 ml of 4 M acetic acid. The main part of the product came off the column in the interval between 13 and 26 ml.

The SAM-containing fractions were evaporated in SpeedVac and dried in a freeze-dryer. A mixture of (S,S)- and (R,S)-SAM diastereomers was obtained, which is in agreement with the earlier described results (Bennett et al., 2017). The purity of the obtained product was controlled by NMR spectroscopy, the concentration was determined by UV spectrophotometry (ε_260_ = 16,000 M^−1^ cm^−1^). The overall conversion rate can be estimated at 50% of the initial SAH. The yield of pure substance after purification was 10%.

^13^C, ^15^N uniformly labelled SAH was obtained from *E. coli* cell line overexpressing the WBSCR27 protein and grown on ^13^C,^15^N M9 medium. The method was based on the propensity of WBSCR27 to be co-purified with the cofactor and isolated in the form of a complex with SAH. The details of this protocol will be published elsewhere. The quality of the obtained ^13^C,^15^N-SAH was confirmed by 1D and 2D NMR spectra (Supplementary Figure S1). The sample contained DTT as an impurity, which does not interfere with the following procedure and was not removed from the product. The concentration of the obtained SAH was measured using UV absorbance at 260 nm (ε_260_ = 16,000 M^−1^ cm^−1^). In total, we obtained 3.2 µmol of ^13^C,^15^N-SAH from 2 L of ^13^C,^15^N M9 medium.

### Cell Lines

Mouse embryonic fibroblast cells NIH3T3 were cultured in DMEM/F12 medium (Gibco), supplemented with 10% FBS (Gibco), 1% Penicillin/Streptomycin (Gibco), and 1% Glutamax (Gibco) at 37°С, 5% CO_2_ and used for all genetic manipulations.

The cell lines created for this study are schematically shown in Supplementary Figure S2. The cell lines A and B were prepared for studying intercellular localization of WBSCR27. The cell line A ectopically expressed fusion of WBSCR27 with far-red fluorescent protein mKate2 on N-terminal (Shemiakina et al., 2012). Similarly, the cell line B ectopically expressed hemagglutinin epitope YPYDVPDYA known as an HA tag (Field et al., 1988). The cell line B together with an endogenous C-terminal WBSCR27-HA fusion (cell line C) were used in co-immunoprecipitation experiments aimed at finding the possible macromolecular partners of WBSCR27. The knockout line (D) containing point mutations in the 2^nd^ exon was used to study the phenotypic consequences of WBSCR27 depletion. To confirm WBSCR27 depletion on the protein level an HA-tag was added to the C-terminus of WBSCR27 in the knockout line (E). Two cell lines F and G were created from the knockout line for proximity labelling in the BioID experiment (Roux et al., 2018). These cell lines ectopically expressed prokaryotic biotin ligase BirA mutant (R118G) from *E.coli* (designated as BirA*), fused to HA and WBSCR27 (HA-BirA*-WBSCR27) or only to HA as the control (HA-BirA*). The details of cloning and constructing these cell lines are provided in Supplementary Data.

### WBSCR27 Localization in the Cell

The intercellular localization of WBSCR27 was verified using cells with ectopic expression of HA or mKate2 (Shemiakina et al., 2012) fusions with WBSCR27. mKate2-WBSCR27 and HA-WBSCR27 expressing cells were seeded on coverslips and kept in the incubator overnight. The next day, the cells were washed 3 times for 5 min with PBST (PBS + 0.1% Triton X-100) and fixed with freshly-prepared 4% paraformaldehyde (in PBS) for 10 min at room temperature. The coverslips were rinsed with PBST (3 times for 5 min), followed by permeabilization with 1% Triton X-100 (in PBS) for 15 min at room temperature and subsequent PBST wash (3 times for 5 min).

The permeabilized coverslips with mKate2-WBSCR27 cells were incubated with 100 mM DAPI in PBS for 5 min at room temperature, followed by washing with PBST (2 times for 7 min). The coverslips were mounted with Mowiol (Sigma) and dried overnight.

The permeabilized coverslips with HA-WBSCR27 cells were blocked with 3% BSA (in PBST) for 1 h at room temperature, followed by incubation with primary anti-HA antibodies (Sigma, 3F10) and then with secondary goat anti-rat Alexa555 conjugated antibodies (Thermo Fisher Scientific) overnight at 4°С in PBST. After washing with PBST (3 times for 5 min), the coverslips were subjected to DAPI staining and mounting as described for mKate2-WBSCR27. Imaging was done with the Nikon Ti-E fluorescence microscope.

### Co-Immunoprecipitation

Cells were cultured in five 15 cm plates to 95% confluency in a DMEM-F12 medium supplemented with 10% FBS, Glutamax, penicillin and streptomycin, and doxycycline hyclate (Sigma) at a concentration of 1 μg/ml at 37°C, 5% CO_2_. The cells were harvested by trypsin, washed twice with 5 ml of PBS and kept frozen at −80°C prior to the immunoprecipitation (IP) experiment.

The frozen cells were resuspended in a lysis buffer [100 mM Hepes-KOH pH 7.5, 150 mM NaCl, 0.05% Triton X-100, 1 mM DTT and complete protease inhibitor cocktail (Roche)]. After centrifugation at 13,000 g for 30 min, the supernatant was transferred to a new tube. For IP, 100 µl of anti HA-beads (Sigma Aldrich) were added to the lysate obtained from 1 g of wet cell mass and incubated overnight at 4°С. After five washes with the lysis buffer, proteins were eluted with a PAGE loading buffer for 5 min at 95°С. The protein eluates were analyzed by PAGE followed by silver staining and Western-blotting. Several bands present exclusively in the samples corresponding to the HA-tagged WBSCR27 were analyzed using MALDI according to the standard protocol (Chugunova et al., 2019).

For cross-linking experiments, the cells were resuspended in 1% formaldehyde (in PBS) and mixed for 7 min at room temperature. The cells were pelleted (500 g, 3 min) and washed twice with 1.25 M glycine (in PBS) to quench the remaining formaldehyde. Next, the cells were lysed as described in the paragraph above. The control sample was subjected to the same protocol, but omitting the formaldehyde addition step.

### BioID Pull-Down

The BioID experiment was performed in accordance with the published protocol (Roux et al., 2018). Three cell lines based on NIH3T3 ΔWBSCR27 were used in the experiment: expressing HA-BirA*-WBSCR27, HA-BirA*, and parental ΔWBSCR27.

Ten 15 cm dishes for each cell line were seeded. Biotin labelling was performed when the cells reached approximately 80% confluency, the medium was changed to a fresh complete medium containing 50 μM biotin and incubated for 16–18 h. In the next stage the medium was completely removed by aspiration, the cells were rinsed twice with 5 ml/dish of PBS, and treated by 600 µl of lysis buffer/dish. The cells were harvested by gentle scraping. After centrifugation we obtained 1–2 g of the cells. The affinity purification of biotinylated proteins was performed using Dynabeads M-280 Streptavidin (Thermo Fisher Scientific).

The eluates from streptavidin beads were treated by trypsin and subsequently analyzed by shotgun proteomics (a technique for identifying proteins in complex mixtures such as cell lysates using a combination of high performance liquid chromatography and tandem mass spectrometry). Mass spectroscopy analysis was performed in triplicates with a Q Exactive HF-X mass spectrometer (Q Exactive HF-X Hybrid Quadrupole-Orbitrap™ Mass spectrometer, Thermo Fisher Scientific, Rockwell, IL, United States). The experimental details were published earlier (Laptev et al., 2020). The obtained raw data were processed using SearchGui (Barsnes and Vaudel, 2018) and PeptideShaker (Vaudel et al., 2015) programs with built-in search engines X! Tandem, MS Amanda, OMSSA, and Comet. Protein sequences of the complete mouse proteome provided by Uniprot (August 2019) were used for protein identification. N-terminal acetylation as well as the oxidation of methionine residues were set as variable modifications for the peptide search. Up to two missed cleavages were allowed for trypsin digestion. The false discovery rates for peptide and protein identifications were set to 1%.

### The Primer Extension Assay

The experiment was performed as described earlier (Lesnyak et al., 2006). Total RNA was purified from NIH3T3 cell lines (WT and ΔWBSCR27) using Trizol reagent (ThermoFisher). The reverse transcription was performed with Maxima Reverse Transcriptase (ThermoFisher) using ^32^P-labelled oligonucleotide complementary to the 28 S rRNA fragment 4,537–4,551. The products of the reverse transcription were separated by electrophoresis in the 10% (w/v) denaturing polyacrylamide gel and visualized by phosphorimagery.

### NMR Spectroscopy

NMR samples at a concentration of 0.2–0.6 mM for ^13^C and/or ^15^N-labelled WBSCR27 and its complex with SAH were prepared in 95% H_2_O/5% D_2_O, 50 mM NaCl, 50 mM sodium phosphate buffer (pH 7.0), 10 mM DTT, and 0.02% NaN_3_. NMR spectra were recorded at 308 K on Bruker AVANCE 600, 700, 800, and 850 MHz spectrometers equipped with a triple resonance (^1^H, ^13^C, ^15^N) room temperature probe (600 MHz), Prodigy probe (700 MHz), and CryoProbe (800 and 850 MHz), or a quadruple resonance (^1^H, ^13^C, ^15^N, ^31^P) CryoProbe (700 MHz). 1D NMR spectra were processed and analyzed using Mnova software (Mestrelab Research, Spain). 2D and 3D spectra were processed by NMRPipe (Delaglio et al., 1995) and analyzed using NMRFAM-Sparky (Lee et al., 2015).

### NMR Structure Determination

Earlier we reported the backbone and side chain signal assignments for the complex SAH-WBSCR27 (BMRB-27417, Mariasina et al., 2018) and for the apo form of the protein (BMRB-27578, Mariasina et al., 2020) and deposited these data in BioMagResBank (https://bmrb.io). This information was used to determine NMR restraints. Backbone φ and ψ dihedral angle restraints were determined from the chemical shift values of the backbone atoms ^13^Cα, ^13^Cβ, ^13^CO, ^1^Hα, ^1^HN, and ^15^N using TALOS+ software (Shen et al., 2009). Two independent sets of residual dipolar coupling (RDC) constants were measured in the nematic phase of a colloidal suspension of filamentous Pf1 phages (Hansen et al., 1998) and in a dilute liquid crystalline medium, consisting of DMPC/DHPC bicelles (Ottiger and Bax, 1999). The RDC values were calculated as a difference of the ^15^N-^1^H splitting values measured in the IPAP-HSQC spectrum (Ottiger et al., 1998) acquired in anisotropic and isotropic conditions. Hydrogen bond restraints were assigned to the amide groups having slow H/D exchange rates and located near carbonyl groups, as identified in the initial set of structures. NOE distance restraints were determined from the ^1^H,^13^C HSQC-NOESY and ^1^H,^15^N HSQC-NOESY spectra measured with a 100 ms mixing time. The initial set of NOE restraints (mainly intra-residue and sequential correlations) was selected manually. The rest of the cross-peaks were assigned using the automatic iterative procedure of spectra assignment/structure calculation implemented in ARIA 2.3 software (Bardiaux et al., 2012). The assignments were further manually verified by multiple steps of structure refinement using the simulated annealing protocol of the CNS 1.21 software package (Brunger et al., 1998). Database values of conformational torsion angle pseudopotentials (Kuszewski et al., 1997) were used during the final cycles of the structure refinement to improve the quality of protein backbone conformation. The structure quality and restraint violations have been analyzed using the CNS tools, Procheck-NMR (Laskowski et al., 1996) and an in-house written NMRest program (Ivanova et al., 2007). The final families of 20 NMR structures of the SAH-WBSCR27 complex and apo-WBSCR27 were selected from 200 calculated conformers in accordance with the lowest-energy criterion and the absence of the residues in the disallowed regions of the Ramachandran map. The restraints used in structure calculations and statistics for the obtained NMR structures are presented in Supplementary Tables S1, S2. Additional details of structure calculations are provided in Supplementary Material. Structure visualization and analysis were carried out using PyMOL (Schrodinger LLC) and Discovery Studio Visualizer (Dassault Systemes Biovia Corp.).

### Relaxation Measurements and Data Analysis

R_1_ and R_2_ relaxation rates and ^1^H-^15^N heteronuclear NOEs for ^15^N uniformly labelled WBSCR27-SAH complex and the apo-form of WBSCR27 were measured at 308 K on a Bruker AVANCE III HD 700 MHz spectrometer. The measured experimental values were analyzed with a model-free formalism using the program RelaxFit written in-house (Polshakov et al., 1999). All the details of the NMR relaxation data collection and analysis are provided in Supplementary Material.

### H/D Exchange

Amide H/D exchange rates in both ^15^N labelled apo-WBSCR27 and complex WBSCR27-SAH were measured using heteronuclear ^15^N–^1^H NMR spectroscopy (at 35°C and pH 7.0) on a Bruker AVANCE Neo 700 MHz spectrometer. The details of the H/D exchange rate measurements and the calculation of the protection factors of the amide H_N_ atoms are given in the Supplementary Data.

### NMR Studies of WBSCR27-Ligand Interactions

NMR experiments were carried out to test the binding of amino acids, nucleosides, and short DNA fragments to WBSCR27. The WBSCR27-SAM complex was prepared using ^15^N-WBSCR27 (0.3 mM) and SAM (1.2 mM) in 320 µl of 90% H_2_O/10% D_2_O.

To test possible binding of amino acids to WBSCR27, a mixture of seven amino acids (Thr, Ser, Arg, Tyr, Cys, Glu, and Lys) in equimolar ratio was prepared. This mixture was added to WBSCR27-SAM samples to obtain molar ratios WBSCR27:SAM:mixture of 1:4:5 and 1:4:10. To test possible interactions of WBSCR27 with the fragments of nucleic acids, we mixed each of nucleosides (guanosine, uridine, cytidine, thymidine) with WBSCR27-SAM separately to obtain molar ratios WBSCR27:SAM:nucleoside = 1:4:10. We additionally prepared a mixture of desoxyoligonucleotide AAA​CCT​CGC​ATT​ACG​AAC​GGC​TCC with the WBSCR27-SAM sample with a ratio of WBSCR27:SAM:DNA = 1:4:1. The purpose of testing an interaction of this arbitrary oligonucleotide with WBSCR27 was to check the possibility of oligonucleotide chain binding by protein. For each sample the ^15^N,^1^H HSQC spectrum was measured at 308 K and 600 MHz.

### Interaction of SAM Epimers With WBSCR27

NMR spectroscopy was used to determine the binding ability of SAM epimers towards WBSCR27. The [methyl ^13^C]-SAM obtained *via* a chemical synthesis from SAH in a concentration of 0.1 mM was used for this purpose. Since the synthetic product is an equimolar mixture of (S,S)- and (R,S)-SAM diastereomers, the concentration of individual components was 0.05 mM. ^13^C,^1^H HSQC spectra were recorded at 308 K and 600 MHz ^1^H frequency for four samples: 1) a free SAM [a mixture of 0.05 mM (S,S)-SAM and 0.05 mM (R,S)-SAM], 2) a free apo-WBSCR27 (protein concentration 0.05 mM), 3) a mixture of 0.05 mM (S,S)-SAM, 0.05 mM (R,S)-SAM, and 0.05 mM WBSCR27 (1:1:1 ratio), and 4) a mixture of 0.05 mM (S,S)-SAM, 0.05 mM (R,S)-SAM, and 0.1 mM WBSCR27 (1:1:2 ratio).

## Results

### WBSCR27 Is Localized in Both Cytoplasm and Nucleus

**FIGURE 1:**
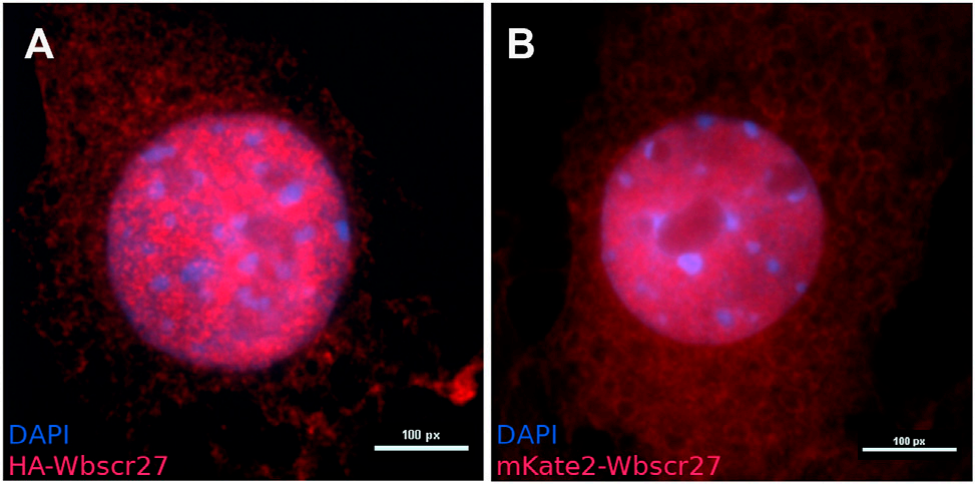
The intracellular localization of WBSCR27 in NIH3T3 cells. **(A)** HA-WBSCR27; **(B)** mKate2-WBSCR27. The signal is present in both the cytoplasm and nucleus. The nucleus was visualized with DAPI.

To determine the intracellular localization of WBSCR27 we inserted HA-WBSCR27 and mKate2-WBSCR27 fusion protein genes under a doxycycline inducible promoter into the NIH3T3 cell line *via* Sleeping Beauty transposase (Mátés et al., 2009). Visualizing both fusion proteins by anti-HA epitope immunocytochemical staining of fixed and permeabilized cells for HA-WBSCR27, and fluorescent microscopy of cells expressing mKate2-WBSCR27 allowed us to reveal both the cytoplasmic and nucleus distribution of WBSCR27 (Figure 1).

### WBSCR27 Apparently Does Not Establish Stable Interactions With Proteins and RNA

To outline the putative partners WBSCR27 protein interacts with, we applied a NIH3T3 cell line with ectopic expression of HA-WBSCR27. After inducing the fusion gene expression by doxycycline, HA-WBSCR27 was immunoprecipitated by an immobilized anti-HA antibody (Supplementary Figure S3A). While a protein of 27 kDa mass identified as HA-WBSCR27 by immunoblotting (Supplementary Figure S3B) was clearly present in the immunoprecipitate, no bands of its putative protein partners were observed. To exclude the possibility that the lack of identifiable partner proteins results from HA-WBSCR27 overexpression (which should lead to a significant decrease in the portion of the complex of WBSCR27 with its potential partner against the background of free protein), or that an N-terminally located HA tag prevents interaction with partner proteins, we used CRISPR/Cas9 directed cleavage and subsequent homologous recombination to create an NIH3T3 cell line with the natural *Wbscr27* gene C-terminally appended with an HA coding part. Immunoprecipitation of WBSCR27-HA from the extracts of the latter cell line (Supplementary Figure S3C) did not lead to the identification of potential WBSCR27 partner proteins.

The homolog of WBSCR27, rRNA MTase WBSCR22, and a number of other MTases form a complex with TRMT112. To specifically address the possibility that TRMT112 might coprecipitate with WBSCR27, we analyzed the eluate after HA-WBSCR27 immunoprecipitation by TRMT112 specific antibodies (data not shown) and found no evidence favoring the interaction between WBSCR27 and TRMT112.

The WBSCR27 MTase might establish only transient contacts with its possible substrates, being disengaged in the process of immunopurification. To this end, we immunopurified ectopically expressed HA-WBSCR27 following the formaldehyde treatment of the cells. Application of the formaldehyde cross-linking to isolate WBSCR27 partner proteins also did not help to identify the interacting proteins due to the absence or negligible yield of covalently crosslinked products (Supplementary Figure S4).

An alternative approach to address the short-lived protein-protein interactions is BioID, a proximity-dependent protein biotinylation *in vivo* by mutant promiscuously active biotin ligase BirA* followed by biotin-affinity capture (Roux et al., 2018). To apply BioID to search for WBSCR27 protein partners we created a cell line expressing HA-BirA*-WBSCR27 fusion and a control cell line expressing HA-BirA*. After labelling was induced by supplementing the cell cultures with biotin, the biotinylated proteins were purified from the cell extracts *via* streptavidine affinity capture and analyzed by shotgun proteomics. While both HA-BirA*-WBSCR27 and HA-BirA* fusion proteins were successfully expressed (Supplementary Figure S5A) and biotinylated endogenous proteins (Supplementary Figure S5B), panoramic proteome analysis of the biotinylated proteins (Supplementary Table S3) did not identify proteins modified specifically by the WBSCR27 protein fusion.

To identify potential RNA partners of WBSCR27 protein we applied PAR-CLIP protocol as described by Gopanenko and co-authors (Gopanenko et al., 2017). The cells of the NIH3T3 line expressing HA-WBSCR27 protein grown in the presence of 4-thiouridine were subsequently subjected to mild UV irradiation to induce RNA-protein cross-linking. After RNA fragmentation and HA-WBSCR27 immunopurification, cross-linked RNA was labelled with γ-[^32^P]ATP and analyzed by gel electrophoresis and autoradiography (Supplementary Figure S6). Despite our efforts, the PAR-CLIP method did not allow identifying an RNA partner/substrate of the WBSCR27 enzyme.

### WBSCR27 Gene Inactivation

To search for potential WBSCR27 substrates we used CRISPR/Cas9 guided WBSCR27 gene inactivation. Biallelic mutations disrupting the WBSCR27 reading frame were introduced to the second exon of the gene. Due to the absence of sufficient specificity of anti-WBSCR27 antibodies (data not shown), we verified the lack of WBSCR27 protein in the knockout cell line by biallelic extension of the *Wbscr27* reading frame with the HA coding region. Disrupting the reading frame of the *Wbscr27*-HA gene resulted in the disappearance of the band stained by anti-HA antibodies (Supplementary Figure S7).

### Probing rRNA Methylating Activity of WBSCR27

For most methylated rRNA nucleotides, the enzymes responsible for their modification are known (Sergiev et al., 2018). The only methylated nucleotide of mammalian ribosomal RNA for which the enzyme responsible for the modification has not yet been identified is the m^3^U4530 of the 28S rRNA (human rRNA numbering). This nucleotide is located in the peptidyl transferase center of the large ribosomal subunit (Sergiev et al., 2018). To validate whether this modification is due to the enzymatic activity of WBSCR27, we carried out a reverse transcription experiment similar to that used to identify a bacterial MTase modifying G2445 of the 23 S rRNA (Lesnyak et al., 2006). The method is based on m^3^U inducing reverse transcription arrest. The experiment showed that the reverse transcription arrest is observed in rRNA from both the WT and WBSCR27 knockout cells (Supplementary Figure S8). These data clearly indicate that WBSCR27 is not responsible for modifying U4530 and, therefore, rRNA cannot be a methylation substrate for this enzyme.

### WBSCR27 Does Not Recognize Fragments of Potential Substrates

The fragment-based lead discovery approach was used to probe the interactions of the possible WBSCR27 substrate fragments with protein. NMR techniques are usually able to detect highly specific interactions of small fragments of a larger ligand with a protein, even in the case of weak binding (Polshakov et al., 2019). We investigated the interactions of small compounds mimicking the fragments of macromolecules, namely: amino acids (Thr, Ser, Arg, Tyr, Cys, Glu, and Lys), nucleosides (guanosine, uridine, cytidine, timidine), and desoxyoligonucleotides with ^15^N-labelled WBSCR27-SAM complex using the methods of heteronuclear NMR spectroscopy. In none of the studied fragments was specific binding observed, leading to a change of the ^1^H and/or ^15^N chemical shifts of the amide groups of certain WBSCR27 amino acids upon adding ligands to the protein.

### WBSCR27 Preferably Binds the Biologically Active (S,S)-SAM Epimer

**FIGURE 2:**
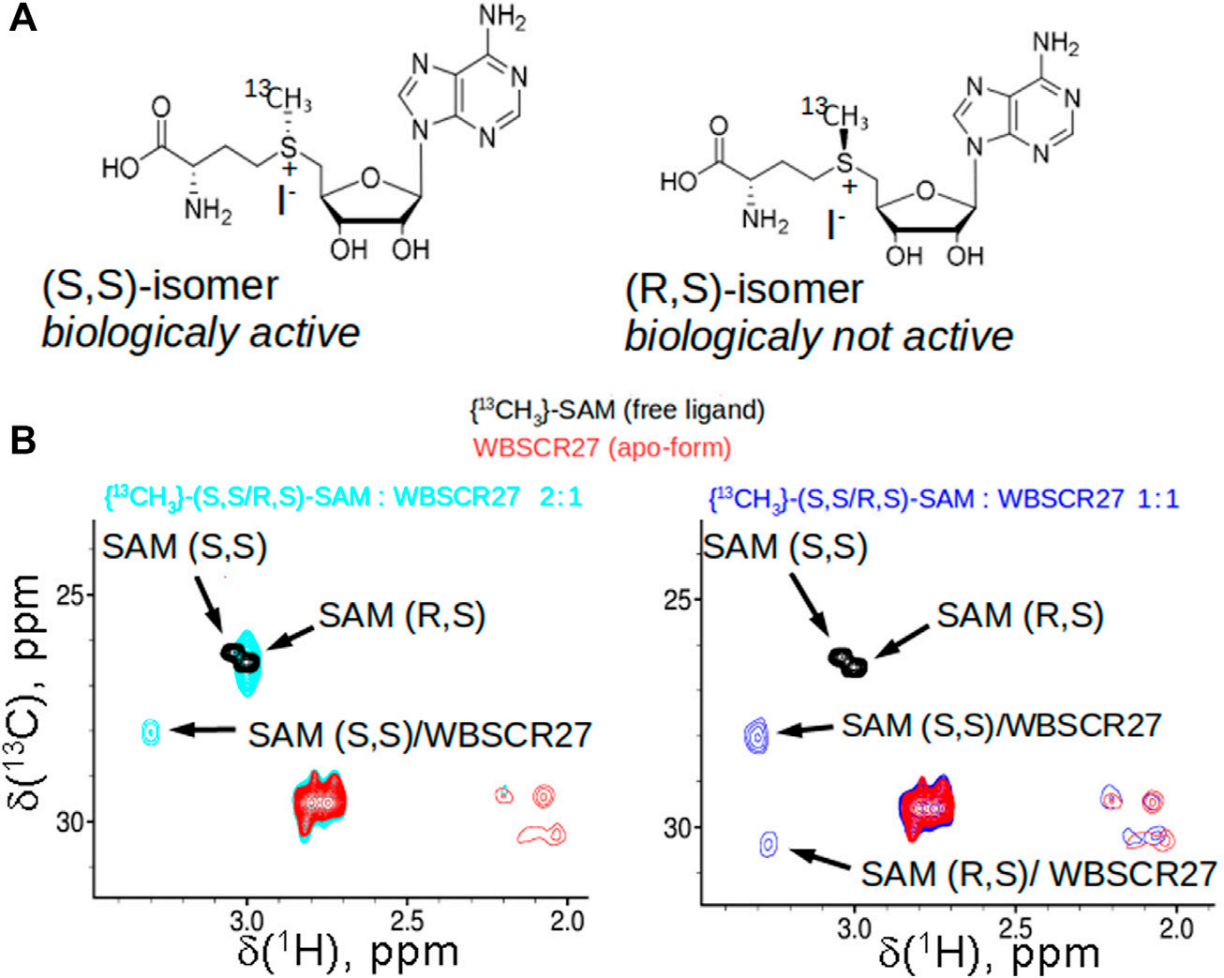
SAM stereoisomers and their binding to WBSCR27. **(A)** Two stable epimers of SAM: biologically active (S,S)-SAM, synthesized enzymatically from ATP and methionine in cells, and (R,S)-SAM, which is obtained as a by-product synthetically. **(B)** Overlay of the fragments of ^13^C,^1^H HSQC spectra of racemic ^13^CH_3_-labelled (R,S/S,S)-SAM (black), apo-WBSCR27 (red) and the mixture of SAM and protein in the ratio 2:1 (cyan, shown in the left panel) or 0.9:1 (blue, shown in the right panel).

The sulfonium atom of SAM represents a chiral center, and both (R) and (S)-epimers are stable (Figure 2A). In living cells, the natural SAM (S,S-epimer) is biosynthesized from L-methionine and ATP by methionine adenosyltransferase (Zhang and Zheng, 2016). Chemically synthesized SAM contains both stereoisomers in equal amounts. We demonstrated by 2D NMR using synthesized [methyl ^13^C]-labelled racemic (S,S/R,S)-SAM that WBSCR27 preferably binds the (S,S) stereoisomer. With an excess of WBSCR27, only the (S,S)-SAM isomer is bound, while the (R,S)-epimer remains in the free form (Figure 2B, left panel). With an increase in WBSCR27 content, the signals of the bound form (R,S)-SAM appear, but their intensity is significantly lower than that of the signals of the (S,S)-SAM-WBSCR27 complex (Figure 2B, right panel). This indicates a significantly lower affinity of the (R,S)-epimer compared with the (S,S)-epimer.

### Solution Structure of Apo-Form of WBSCR27 and Its Complex With S-Adenosyl-L-Homocysteine

Earlier, we found that both SAM and SAH strongly bind to WBSCR27 (Mariasina et al., 2020). Relatively small changes in the chemical shifts of the signals of the residues in the binding site of these two ligands indicate that the structure of WBSCR27-SAM and WBSCR27-SAH complexes is similar (Mariasina et al., 2020). At the same time, bound SAM rapidly (several hours) decomposes to SAH; as a result, only the WBSCR27-SAH complex remains sufficiently stable to measure a series of heteronuclear NMR spectra. To determine the structures of the complex WBSCR27-SAH and the protein in apo-form traditional heteronuclear NMR and restrained molecular dynamics techniques were used. To assign the signals of bound SAH and protein-ligand NOEs, the NMR spectra for the complexes of ^15^N-WBSCR27 with ^13^C-labelled and unlabelled SAH were measured and compared. Several protein-ligand NOEs were also identified in NOESY spectra of the complex of ^13^C,^15^N-WBSCR27 with unlabelled SAH. In total, 21 protein-ligand NOEs were used in structure calculation (Supplementary Table S1).

**FIGURE 3:**
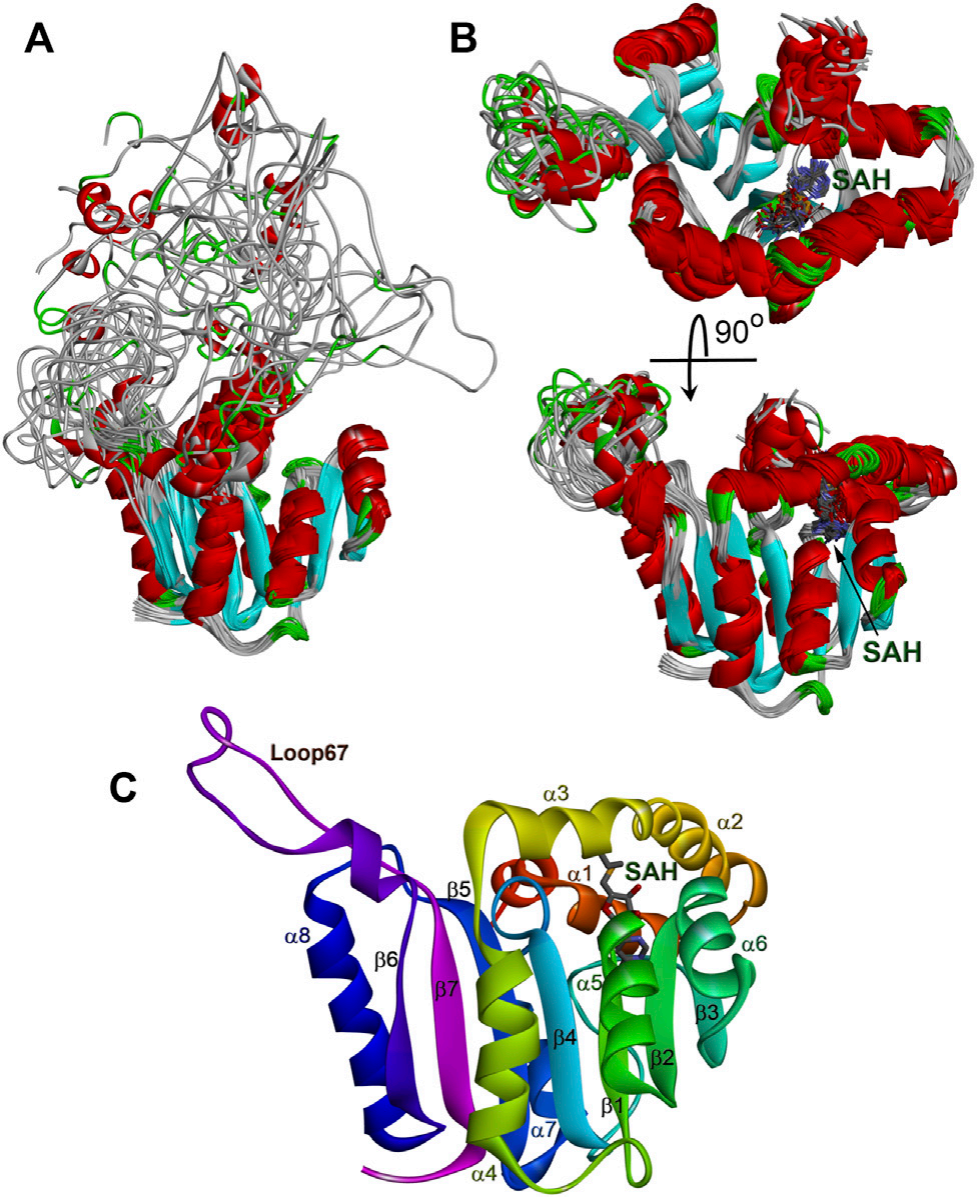
Structure of WBSCR27 in apo-form **(A)** and in complex with SAH **(B,C)**. Families of 20 NMR conformers **(A,B)** and rainbow colored structure of WBSCR27-SAH complex with labelled elements of the protein structure are shown.

**FIGURE 4:**
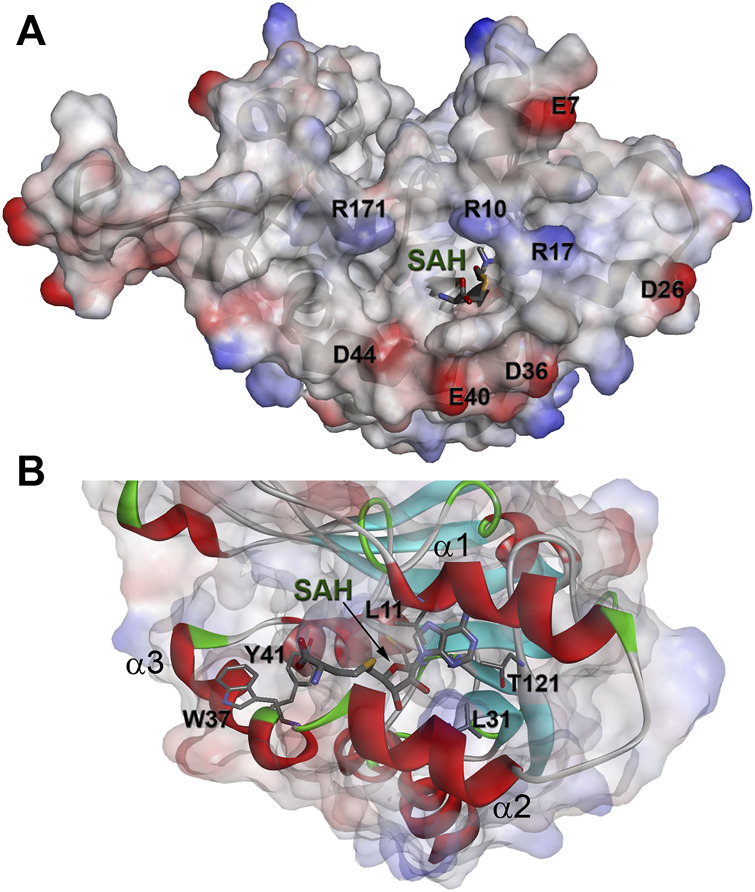
The binding site of SAH in complex with WBSCR27. **(A)** Electrostatic surface potential of the protein. **(B)** Amino acids of WBSCR27 participating in the interaction with SAH.

The solution structure of the WBSCR27 apo-form (Figure 3A) shows that the first 51 amino acid residues of the protein as well as the loop between residues 204 and 228 (loop67 in Figure 3C) are unstructured. SAH binding to the protein puts in order the N-terminal protein fragment by forming three well-structured α-helices (α1–α3) and a short helical part in the first nine residues (Figure 3B). The structure of WBSCR27 has a canonical Rossman fold, typical of most of the Class I MTases (Figure 3C). The protein core consists of seven β-strands (β1–β7) surrounded by five α-helices (α4–α8, Figure 3C). SAH binds to the residues on the tips of three β-strands (β1–β3) and strengthens these interactions by several hydrophobic and electrostatic contacts with the amino acid residues in the helices α1, α2, and α3 (Figure 4). The purine fragment of SAH binds predominantly to the amino acid residues of the protein β-core, while the methionine fragment interacts exclusively with the residues of helices α1–α3. The position of SAH methionine fragment is determined less precisely. The pairwise RMSD of the coordinates of the heavy atoms of the methionine fragment of SAH in the final family of structures is 2.3 ± 0.5 Å. For the adenosine fragment of SAH this value is 2.0 ± 0.6 Å. This may be due to higher mobility of the first three α-helices relative to the protein core.

### WBSCR27 Backbone Dynamics

**FIGURE 5:**
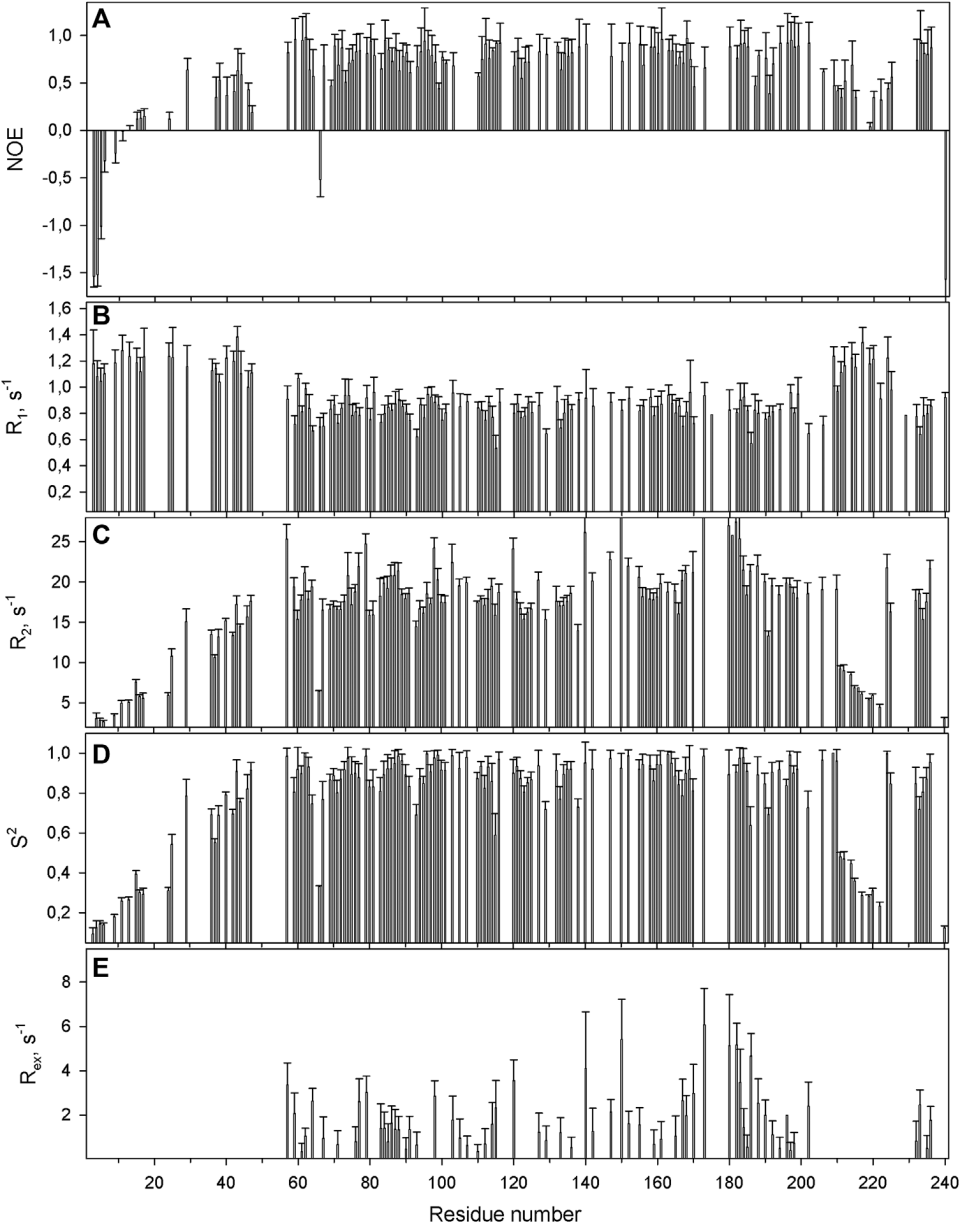
The relaxation parameters of the amide ^15^N nuclei of each residue of the apo-form of WBSCR27, measured at 16.3 T (700 MHz proton resonance frequency) and 308 K. **(A)** The heteronuclear ^15^N,^1^H-steady-state NOE values. **(B)** The longitudinal relaxation rate *R*_*1*_ (s^−1^). **(C)** The transverse relaxation rate *R*_*2*_ (s^−1^). **(D)** The order parameter *S* ^*2*^ determined by model-free analysis. **(E)** Chemical exchange *R*_*ex*_ contributions to the transverse relaxation rates (s^−1^).

**FIGURE 6:**
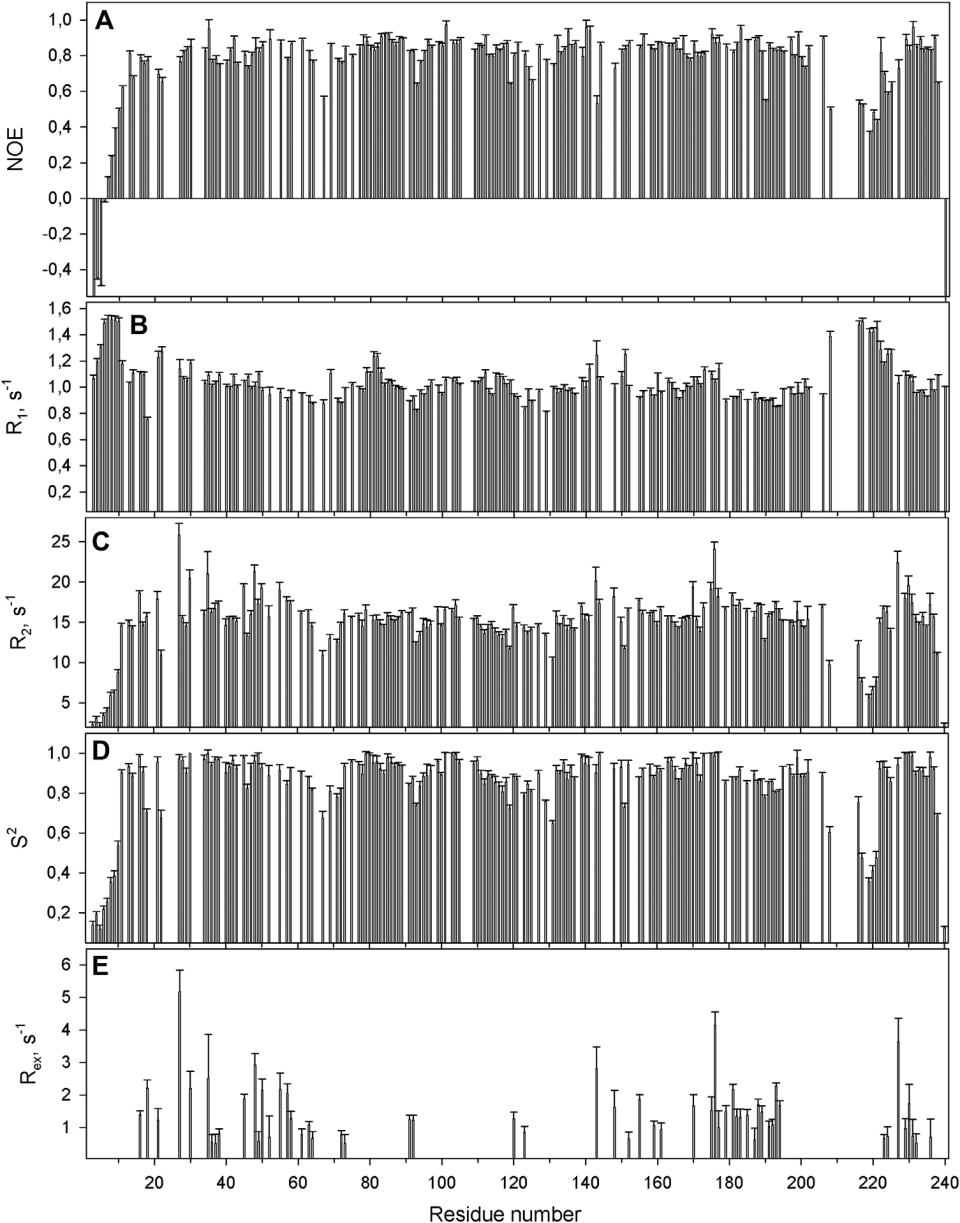
The relaxation parameters of the amide ^15^N nuclei of each residue of the SAH-WBSCR27 complex, measured at 16.3 T (700 MHz proton resonance frequency) and 308 K. **(A)** The heteronuclear ^15^N,^1^H-steady-state NOE values. **(B)** The longitudinal relaxation rate *R*_*1*_ (s^−1^). **(C)** The transverse relaxation rate *R*_*2*_ (s^−1^). **(D)** The order parameter *S* ^*2*^ determined by model-free analysis. **(E)** Chemical exchange *R*_*ex*_ contributions to the transverse relaxation rates (s^−1^).

The protein backbone dynamics of WBSCR27 in both the apo-form and the complex with SAH was investigated by analyzing ^15^N relaxation experiments and hydrogen-to-deuterium (H/D) exchange rates of the amide protons. The values of the overall rotational correlation time τ_c_, calculated from the ^15^N *T*_1_ and *T*_2_ data measured at 308 K (Kay et al., 1989), are 12.5 ± 0.2 and 10.4 ± 0.3 ns for the apo-form and the WBSCR27-SAH complex, respectively. The differences in the τ_c_ values for the apo-form and the complex appear to reflect the distinctions in the shape of the protein molecule. The protein globule is apparently more compact in the case of the WBSCR27-SAH complex, while the long unstructured N-terminal tail in the apo-form slows down protein tumbling. The experimentally measured ^15^N relaxation parameters were interpreted using the model-free formalism (Lipari and Szabo, 1982) with extension to include chemical exchange contributions *R*_*ex*_ to the transverse relaxation rates (Clore et al., 1990) (Supplementary Material for details). Figure 5 (for the apo-form of WBSCR27) and Figure 6 (for WBSCR27-SAH) show the measured ^15^N relaxation parameters *R*_*1*_, *R*_*2*_, and NOE with the calculated order parameters *S* ^*2*^ and *R*_*ex*_ values, plotted against the corresponding residue numbers. The mobility of the WBSCR27 backbone in the apo-form is significantly higher than that in the complex with SAH, which agrees well with the observed results of the structural studies.

**FIGURE 7:**
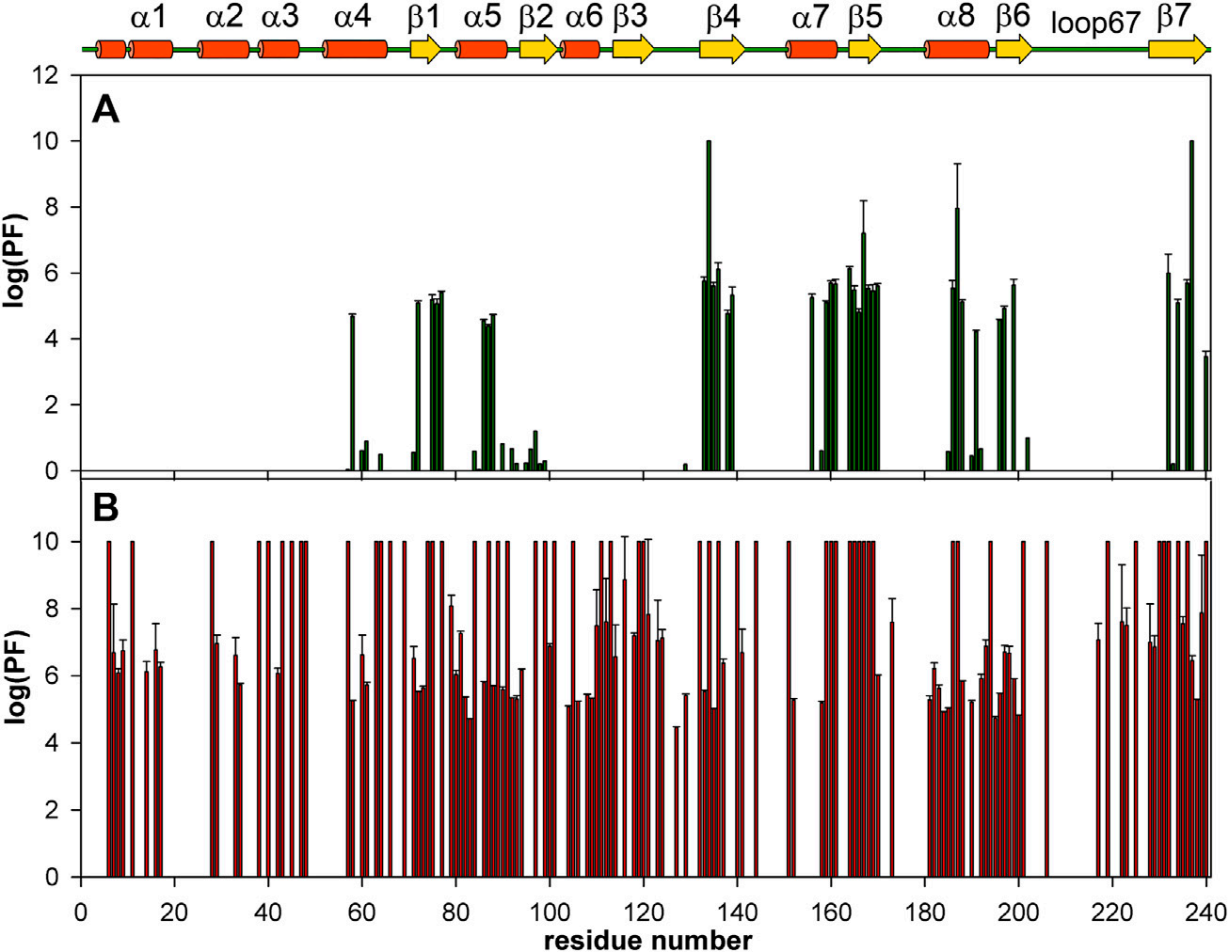
Bar charts showing the amide HN protection factors of the apo-WBSCR27 **(A)** and complex WBSCR27-SAH **(B)**. The elements of protein secondary structure identified for WBSCR27-SAH solution structure are shown on the top.

Figure 7 shows the distribution of the protection factors PF for the amino acid residues of the apo-form of WBSCR27 and the WBSCR27-SAH complex, as analyzed from the measured proton-to-deuterium exchange rates. Binding of SAH leads to a significant increase in the PF of most amino acid residues of WBSCR27, which reflects the slowing down of high-amplitude protein backbone motions upon the ligand binding and the strengthening of the hydrogen bond network within the protein molecule.

## Discussion

Dissecting the WBSCR27 structure and delineating its functions may pave the way to understanding the molecular mechanisms underlying the clinical manifestations of WBS. In its turn, this may contribute to developing clinical interventions aiming to compensate for the symptoms of this genetic disease.

### Binding of SAH Causes Structuration of the N-Terminal Tail of WBSCR27 and General Tightening of Protein Structure in Solution

**FIGURE 8:**
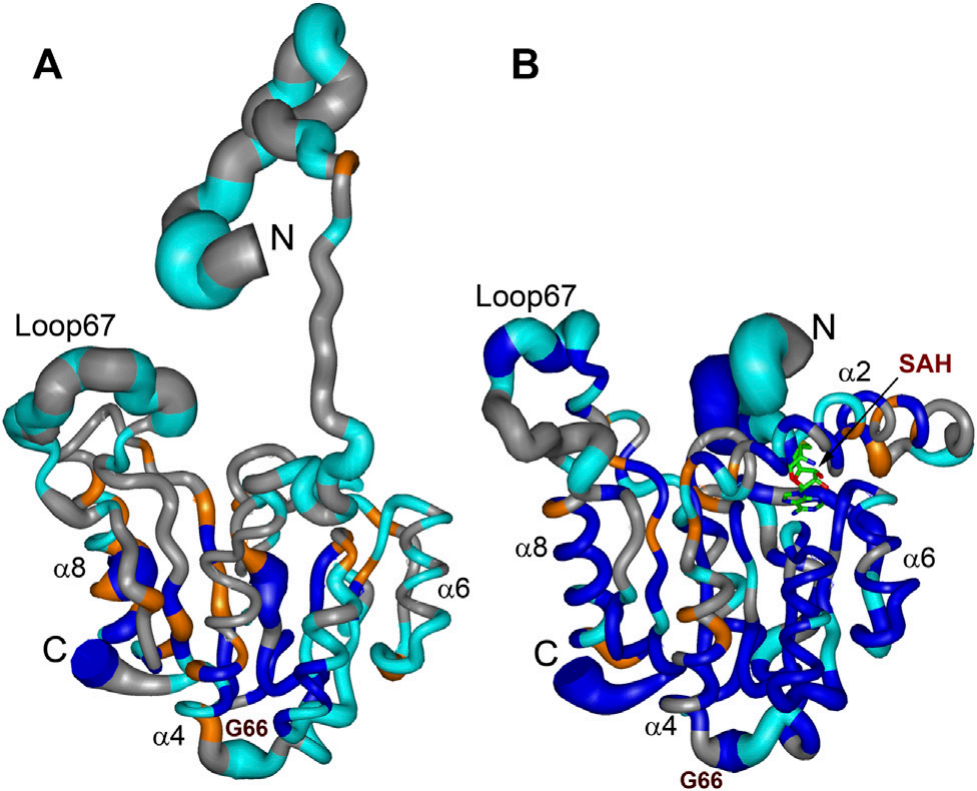
Summary of dynamic and conformational behaviour of the WBSCR27 in apo-form **(A)** and in complex with SAH **(B)**. Representative NMR models of apo-WBSCR27 and complex SAH-WBSCR27 are shown according to protein mobility in a broad time scale. The thickness of the chain is proportional to the value (1 − S^2^) representing the extent of the local amplitude of protein backbone motion in ps-ns time scale. Fragments of the protein backbone containing amino acids undergoing conformational exchange in ms time scale (with values R_ex_ exceeding 2 s^−1^) are colored orange. Protein backbone fragments with residues having high values of protection factors determined from the analysis of hydrogen-to-deuterium HN exchange rates are colored blue. Residues for which ^15^N relaxation data could not be obtained (proline residues and those with overlapped HN signals) are shown in gray. Representative secondary structure elements and SAH molecule are labelled.

Comparing structures of the WBSCR27 apo-form and its complex with SAH indicates the formation of three additional α-helices at the N-terminal tail of the protein upon the cofactor binding (Figure 3). In the apo-form the first 50 residues forming these helices turn out to be disordered. These structural observations are clearly confirmed by the results of the protein backbone dynamics studies (Figures 5–8). For many residues of the apo-WBSCR27 the order parameters of amide NH bonds, determined *via* the ^15^N relaxation measurements, are much lower than the corresponding values for the WBSCR27-SAH complex (Figures 5, 6). These results indicate a high amplitude backbone motion of the apo-form of the protein in a time scale from ps to ns. There are also significant differences in the rates of backbone motions occurring in the ms time scale and characterized by the conformational exchange. The protein fragments which contain residues participating in conformational exchange are colored in orange in Figure 8.

The amplitude of the fast protein backbone motions, as well as the conformational transitions occurring in the ms timescale, are much greater for the case of the apo-form than for the WBSCR27-SAH complex. This difference in protein dynamics is observed not only for the first 50 amino acid residues unstructured in the apo-form, but also for the well-structured protein core. For example, the fragments of β4, β5, and α8 are highly mobile in the apo-form. Loop 67 remains highly mobile, both in the apo-form and in the complex. This loop is likely to be involved in recognizing the substrate molecule, and following its binding with helices α1–α3, loop 67 fixes this interaction, after which its mobility should disappear. Notably, the mobility of the G66 residue is high in both forms: the apo-form and the WBSCR27-SAH complex, the magnitude of the ^15^N-^1^H NOE of the amide group of this residue is negative. However, the mobility of this residue located at the apex of the loop between α1 and β1 is unlikely to play any functional role.

The differences in the amplitude of the slow protein backbone motions, occurring on a time scale from minutes to hours and determined from analyzing the H/D exchange, are even more obvious when we compare the apo-form and the protein-ligand complex. Relatively high values of amide NH protection factors for the apo-form of WBSCR27 are observed only for the very central part of the protein core (Figures 7, 8). Interestingly, all the amide groups of the residues from outer β-strand β3 and helices α4 and α6 are unprotected in the apo-form of the protein indicating a high mobility of these elements of the secondary structure. After SAH binding, almost the whole protein molecule, except for loop regions, turns out to be well protected from exchanging amide protons with water. This follows from large values of the protection factors of the corresponding NH groups (Figure 8).

### Cofactor Binding Site

Helices α1–α3 in the WBSCR27-SAH complex surround the SAH molecule and partially form its binding site. These three helices also form a binding site for the potential substrate of the methylation reaction, catalyzed by WBSCR27. The adenosine fragment of SAH binds to the tips of the three strands β1, β2, and β3, while the methionine chain is positioned between the helices α2 and α3. The backbone carbonyl groups and amide hydrogens of the residues 100, 101, and 121 form several hydrogen bonds with the adenosine fragment. The side chains of the residues L11, L31, A77, and T121 form hydrophobic interactions with the adenine moiety of the SAH. In the region of the methionine fragment, there are also two aromatic residues, Y41 and W37. If the role of Y41 is most likely related to interacting with the carboxyl or amino group of the SAH methionine fragment, then W37 may participate in the interaction with a potential substrate fragment. It may, for instance, hold the aromatic base of RNA or DNA by stacking interaction in the position favorable for methylating this nucleotide or the neighboring one. This possibility is evidenced by the outward orientation of the side chain of W37 from the protein core, and its proximity to the sulfur atom of the SAH methionine residue.

### Comparing WBSCR27 Structure With Other Class I MTases

**FIGURE 9:**
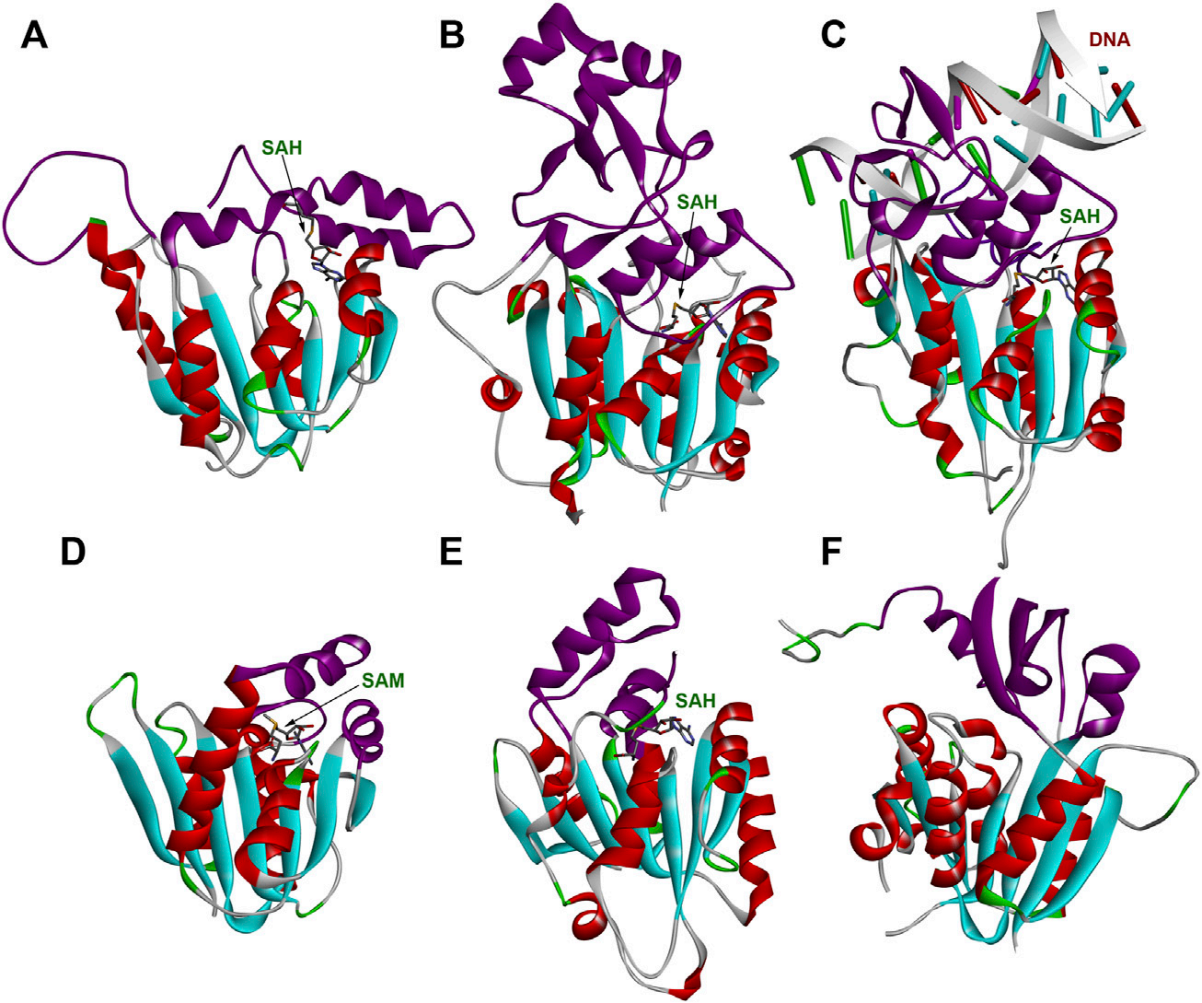
Structures of several MTases. **(A)** WBSCR27 in complex with SAH (PDB id 7QCB); **(B)** *S. pombe* tRNA^Asp^ MTase DNMT2 in complex with SAH (PDB id 6FDF); **(C)** human DNA MTase DNMT3A in complex with SAH and DNA fragment (PDB id 6BRR); **(D)** *S. cerevisiae* rRNA MTase Bud23 (PDB id 4QTU); **(E)** human tRNA^His^ MTase BCDIN3D in complex with SAH (PDB id 6L8U); **(F)** human glycine MTase (PDB id 2AZT). Rossmann-fold of the enzymes’ core is colored in cyan (β-strands) and red (α-helices). Substrate-binding domains are colored purple.

The structure of WBSCR27 represents a classical Rossmann fold (Figure 3C), typical of all Class I SAM-dependent MTases. More than 120 various members of this enzyme family were classified (Martin and McMillan, 2002). They have different methylation substrates and very little sequence identity, but a highly conserved structural fold (Figure 9) and a β-sheet core, formed by seven β-strands (Figure 9). There are some variations in the number, length and orientations of α-helices surrounding this β-sheet, but they are still rather conservative in their structure. The greatest differences, as expected, are observed in the structure of the substrate-binding regions (colored purple in Figure 9), although the substrate molecule can also interact with the residues in the core region of the protein. The substrate-binding domain should ensure the selectivity of the substrate molecule binding and its correct positioning relative to the methyl group of the co-factor. However, the variability in the structure of the substrate-binding domain is great even for one type of substrate. Figures 9B,D,E show the structures of RNA MTases with significantly different substrate-binding fragments. Notably, there are two or three α-helices in the proximity to the SAM binding site in RNA MTases. A similar topology is observed in the case of DNA MTases (Figure 9C). The topology of the substrate-binding domain for small molecule methylation MTase (the structure of glycine MTase is shown in Figure 9F as an example, Luka et al., 2007) is markedly different. Based on these structural considerations, a nucleic acid would be the most likely substrate for WBSCR27, but other options cannot be ruled out.

One of the closest sequence homologs of WBSCR27 is a human protein WBSCR22 and its yeast ortholog Bud23 (Mariasina et al., 2018). WBSCR22 is a 18S rRNA MTase involved in pre-rRNA processing and ribosome 40 S subunit biogenesis (Haag et al., 2015). WBSCR22 has an interaction partner—the protein TRMT112 which is vital for the functional activity of this MTase in mammalian cells (Õunap et al., 2015). The known 3D structure of the complex of Bud23 with TRMT112 (Létoquart et al., 2014) allows comparing the TRMT112-binding interface on the surface of Bud23 with the similar area of the molecular surface of WBSCR27 (Supplementary Figure S9). The patterns of electrostatic potentials on the surface of Bud23 and WBSCR27 are quite different, and it is unlikely that TRMT112 can be the functional partner of WBSCR27. However, we tested this hypothesis using co-immunoprecipitation and antibody staining for TRMT112. The experimental data obtained confirm the conclusion that these proteins do not interact with each other.

The protein folding topology and three-dimensional structure of WBSCR27 are similar to those of RNA MTase GidB (Romanowski et al., 2002). Notably, for GidB, as well as for WBSCR27, the enzyme function and methylation substrate were initially unknown. At the same time, establishing the three-dimensional structure of this MTase accelerated identifying the methylation substrate. This enzyme (alias RsmG) was recently shown to be responsible for N7 methylation in G527 of 16 S bacterial rRNA (Abedeera et al., 2020).

### Possible Substrates of WBSCR27 and Its Potential Function

While our work has not yielded WBSCR27 substrates and partners, the negative result of this kind is also informative potentially narrowing down the range of possibilities for future research. First and foremost, WBSCR27 seems not to form any stable interaction with a substrate, unlike MTases functioning as the molecular switches in ribosome assembly, i.e., bacterial KsgA (Connolly et al., 2008) or mammalian METTL15 (Laptev et al., 2020). A number of MTases responsible for modifying the translation apparatus components and other substrates form a stable complex with the TRMT112 protein (Zorbas et al., 2015; Metzger et al., 2019; van Tran et al., 2019; Yang et al., 2021), while mRNA specific MTase METTL3 forms a stable complex with METTL14 (Liu et al., 2014; Wang et al., 2014) and WTAP (Ping et al., 2014). The results of this study disfavor the scenario that WBSCR27 forms a stable functional complex with other proteins. WBSCR27 is likely to be a standalone MTase only transiently interacting with its substrate. Moreover, WBSCR27 is unlikely to catalyze protein methylation, otherwise there must be a target protein that would be found in one of the experiments described above.

Our results do not exclude that WBSCR27 possibly participates in the methylation of a small molecule, whose chemical properties do not allow it to be identified in cell lysates by NMR methods. An overarching theory unpacking WBSCR27 functions is still ahead, and we hope that determining the three-dimensional structure of this enzyme in the apo-form and in the form of a complex with SAH will help achieve this goal.

## Data Availability

The datasets presented in this study can be found in online repositories. The names of the repository/repositories and accession number(s) can be found in the article/Supplementary Material.
