# Supplementary material for: Williams-Beuren Syndrome Related Methyltransferase WBSCR27: From Structure to Possible Function
Source: Front Mol Biosci. 2022 Jun 15;9:865743. doi: 10.3389/fmolb.2022.865743 (PMC9240639; doi:10.3389/fmolb.2022.865743)
Supplement: Supplementary file 1 [file DataSheet1.PDF]

frontiers

Supplementary Material

1 Supplementary Data

Cloning and constructing cell lines

To create cell lines with ectopic expression of the N-terminal fusions mKate2-WBSCR27 and HA-WBSCR27 we cloned cDNA coding mouse WBSCR27 into the pSBTet-Neo plasmid (Kowarz et al., 2015). In order to append the N-terminus of WBSCR27 with an HA tag, we amplified the plasmid with primers containing overhangs coding for the HA-tag and circularized the obtained product by self-ligation. mKate2 was cloned to the N-terminus of WBSCR27 by restriction-free cloning using mKate2 coding sequence with flanking regions complementary to the pSBTet-Neo HA-WBSCR27 as a megaprimer. The oligonucleotides used for the creation of fusions HA-WBSCR27 and mKate2-WBSCR27 are listed in Table S4. For stable expression of HA-WBSCR27 and mKate2-WBSCR27 fusions, the NIH3T3 cell line was co-transfected by pSBTet-Neo plasmids containing the respective insert with pCMV(CAT)T7-SBX100 plasmid (Mátés et al., 2009) by Lipofectamine 3000 (ThermoFisher Scientific) according to the manufacturer's instructions. The stable cell lines were obtained after 7 days of G418 selection.

For insertion of a C-terminal HA-tag to the endogenous *Wbscr27* gene we cloned the sgRNA targeting stop codon region of the gene into pX458 vector (Ran et al., 2013). The pUC19 plasmid containing the HA-tag with the homology arms was used for co-transfection of the NIH3T3 cell line. Success of biallelic tagging was validated with junction PCR, Sanger sequencing, and immunoblotting. The oligonucleotides used for insertion and validation of the C-terminal tagging of WBSCR27 are listed in Table S5.

To generate an NIH3T3 cell line with inactivating frameshift mutations of WBSCR27 in the 2nd exon, the appropriate sgRNAs (Table S6) were cloned into the pX458 vector. The plasmids obtained were used for transfection of NIH3T3 cell line by Lipofectamine 3000 according to the manufacturer's instructions. Transfected cells were separated into single cells by a FACSAria III cell sorter which were placed into individual wells of 96 well plates. Monoclonal cell lines obtained were analyzed by target genome region amplification and sequencing. Cell lines containing a reading frame disruption on both alleles in the 2nd exon were used for further experiments. Attempts to approve the knock-outing by western-blotting with four different anti-WBSCR27 antibodies were performed. Unfortunately, neither antibodies raised against recombinant WBSCR27, nor the ones obtained against peptide mimicking loops of WBSCR27, recognized the endogenous protein. To validate the absence of the WBSCR27 protein in a cell line with knockout on the 2nd exon of WBSCR27, we HA-tagged WBSCR27 at the C-terminus (the experimental procedure described in detail in the previous section). The disappearance of anti-HA stained band corresponding to the WBSCR27-HA observed using anti-HA antibodies (Sigma, 3F10) enabled us to validate depletion of WBSCR27 at the protein level.

The plasmid pSBbi-GN HA-BirA*-WBSCR27 for BioID was created by the following way. The BirA* coding region was amplified from the BirA*-eGFP-Myc plasmid using primers containing SfiI-sites for the subsequent insertion into pSBbi-GN vector (Addgene #60517). An HA-tag and a second SfiI-site was added at the next step with PCR. *Wbscr27* was amplified from the previously

generated plasmid using primers containing BirA*-template matching overhangs. Restriction-free cloning was used to join *Wbscr27* and HA-BirA* coding parts. Primers used for *Wbscr27*-BirA* construction are listed in Table S7.

The control plasmid carrying HA-BirA* was prepared from pSBbi-GN HA-BirA*-WBSCR27 by excision of the *Wbscr27* coding region. For stable expression of HA-BirA*-WBSCR27 or HA-BirA*, the prepared pSBbi-GN plasmids with pCMV(CAT)T7-SBX100 were used for co-transfection of the NIH3T3 ΔWBSCR27 cell line by Lipofectamin 3000 according to the manufacturer's instructions. The stability of expression was assessed after 7 days of selection in G418-containing medium.

NMR restraints

Interproton distance constraints for the apo-form of the WBSCR27 and SAH-WBSCR27 complex were obtained from NOEs measured in 3D ^{15}N-^{1}H and ^{13}C-^{1}H HSQC NOESY spectra recorded in H_2O and D_2O. The full set of restraints used in structure calculation of the SAH-WBSCR27 complex contained 2466 NOEs, 232 hydrogen bonds, 344 dihedral angles and 303 RDCs measured in two anisotropic media (in 5% w/v liquid crystalline bicelle medium of 3:1 DMPC/DHPC (Tjandra and Bax, 1997) and in solution of filamentous Pf1 phages in concentration of ~25 mg/ml (Trempe et al., 2002). The number and distribution of restraints for SAH-WBSCR27 structure calculation are given in Table S1 and illustrated in Fig. S10b. Protein-ligand NOEs were assigned in the NOESY spectra using the information about the chemical shifts of SAH in the bound state (Table S8), which were obtained from the analysis of the NMR spectra of the complex of ^{13}C-SAH with WBSCR27. A total of 21 protein-ligand NOEs were used in structure calculations, most of which belong to the adenosine fragment of SAH.
The higher mobility of the protein backbone in the apo-form of WBSCR27 (see main text) makes the quality of NOESY spectra worse than that of the SAH-WBSCR27 complex. Therefore, the initial set of NOEs for the apo-WBSCR27 structure calculation was extracted from the list of NOEs of the complex for the pairs of the residues in which chemical shifts do not change upon transition from the apo-form to the complex (residues 59-70, 85-96, 106-117, 127-145 and 146-240). These NOEs were verified in the 3D ^{15}N-^{1}H and ^{13}C-^{1}H HSQC NOESY spectra of apo-WBSCR27 and complemented by the NOEs from the protons of missing residues. Such an approach (restrained docking method) has been approved earlier for structure calculation of the complexes of the same protein with different ligands (Polshakov et al., 1999). Distance restraints were further verified during the multiple steps of structure refinement. The final set of restraints used in the structure calculation of apo-WBSCR27 contained 2243 NOEs, 122 hydrogen bonds, 295 dihedral angles and 143 RDCs measured in solution of filamentous Pf1 phages. The number and distribution of restraints for the apo-form of WBSCR27 structure calculation are given in Table S2 and illustrated in Fig. S10a.

NMR structure calculations

The structure calculation was performed by a simulated annealing protocol carried out in Cartesian coordinate space using the CNS 1.2 (Brunger et al., 1998). Database values of conformational torsion angle pseudopotentials (Kuszewski et al., 1997) were introduced at the final stages of structure refinement. The final force constants were as follows: NOE restraints, 75 $kcal \cdot mol^{-1} \cdot Å^{2}$; dihedral angle restraints, 200 $kcal \cdot mol^{-1} \cdot rad^{2}$; RDCs, 30 $kcal \cdot mol^{-1} \cdot Hz^{2}$. The restraint violations were monitored after each cycle of refinement by the in-house program NMRest (Polshakov et al., 1999). Violated restraints were checked against corresponding spectral data and subsequently corrected or declined. The structure

quality was analyzed using the Procheck-NMR software (Laskowski et al., 1993). The best 20 structures out of 200 (with respect to the minimum restraints violation value criterion and Ramachandran plot statistics, see Fig. S11) were accepted as the final ensemble for each protein conformer (apo-form of WBSCR27 and its complex with SAH). Structure visualization and analysis were carried out using the InsightII software package (Accelrys Software Inc.), PyMol (DeLano Scientific LLC) and Discovery Studio Visualizer v. 20 from Dassault Systemes Biovia Co (San Diego, CA, USA).

NMR Relaxation analysis

R_1, R_2 and ^{1}H-^{15}N heteronuclear NOE data sets of ^{15}N uniformly labelled WBSCR27 in complex with SAH were collected at 308K on a Bruker Avance III 700 MHz spectrometer with a z-gradient quadruple resonance (^{1}H, ^{13}C, ^{15}N, ^{31}P) CryoProbe. Analogous relaxation experiments carried out on ^{15}N uniformly labelled apo-form of WBSCR27 were collected at 308K on a Bruker Avance Neo 700 MHz spectrometer with a z-gradient Prodigy triple resonance (^{1}H, ^{13}C, ^{15}N) TCI CryoProbe. The delays for the R_1 relaxation rate experiments were 0.1, 0.15, 0.2, 0.25, 0.3, 0.35, 0.4, 0.45, 0.5, 0.58, 0.64, 0.8, 1.0, 1.3, 1.8 and 2.5 s for SAH-WBSCR27, and 0.01, 0.1, 0.2, 0.3, 0.5, 1.0, 2.0 and 3.0 s for the apo-WBSCR27. The delays for the R_2 relaxation rate experiments were 0, 17, 33.9, 50.9, 67.8, 84.8, 101.8, 118.7, 135.7, 152.7, 169.6, 186.6, 203.5, 237.5, 271.4, 305.3 for the SAH-WBSCR27, and 17, 33.9, 50.9, 67.8, 84.8, 101.8, 118.7 ms for the apo-WBSCR27. The excitation time for ^{1}H in the ^{1}H-^{15}N heteronuclear NOE experiments was 6.0 s, relaxation delays in R_1 and R_2 experiments were 4 s. All the spectra were processed using NMRPipe software (Delaglio et al., 1995). The nonlinear fitting of the integrated peak volumes and calculations of their standard deviations were accomplished using the nlinLS procedure. The values of R_1 and R_2 were calculated from the lists of peak volumes using the in-house program RelaxFit (Polshakov et al., 1999). The standard deviations of the ^{15}N-^{1}H NOE values were calculated using the RMS noise of the background regions (Farrow et al., 1994) and were further verified and corrected using two independently collected experimental data sets. The analysis of the R_1, R_2, and ^{1}H,^{15}N-NOE values for SAH-WBSCR27 and apo-WBSCR27 was carried out using a model-free formalism and the program RelaxFit written in-house (Polshakov et al., 1999).

Calculation of amide NH protection factors

Amide NH exchange rates were determined in a series of ^{1}H-^{15}N HSQC spectra recorded as pseudo-3D experiments after dissolving the lyophilized samples of SAH-WBSCR27 or apo-WBSCR27 in D_2O. Acquisition of the first spectrum was started ~10 minutes after dissolving the protein sample. 46 and 19 HSQC planes were collected for SAH-WBSCR27 and apo-WBSCR27, correspondingly, with time steps every 12 minutes. Rate constants of the H/D exchange k_{obs} for individual NH signals were calculated from the time course of their volumes, measured by the non-linear fitting of the corresponding line shapes using the nLinLS procedure from the NMRPipe package (Delaglio et al., 1995). Calculations of the k_{obs} values were carried out in the RelaxFit program written in-house (Polshakov et al., 1999). Errors in measured k_{obs} values were estimated from their standard deviations obtained in the non-linear fitting. An EX2 mechanism for H/D exchange was assumed to be valid at pH 7.0 (Polshakov et al., 2006). Protection factors PF for the individual amide hydrogens were calculated as ratios of intrinsic (k_{int}) and observed (k_{obs}) exchange rate constants (Molday et al., 1972). Values of k_{int} were calculated as described by Bai and co-workers (Bai et al., 1993) using the corresponding module of the program RelaxFit.

2 Supplementary Figures and Tables

2.1 Supplementary Tables

Supplementary Table 1. Statistics for the ensemble of the calculated 20 structures of the SAH-WBSCR27 complex.

A. Restraints used in the structure calculation

Total distance restraints	2698	Total dihedral angles	344
Long range (|i-j| > 4)	471	Phi (ϕ)	172
Medium (1 < |i-j| < 5)	317	Psi (ψ)	172
Sequential (|i-j| = 1)	691		
Intraresidue	966	Total RDCs	303
Protein-ligand NOEs	21	DMPC/DHPC	135
Hydrogen bonds	232	Phages Pf1	173

B. Restraint violations and structural statistics (for 20 structures)

No NOE or dihedral angle violations are above 0.5 Å and 5° respectively.

Average RMSD	<S>[a]	S_{rep}
From experimental restraints		
Distance (Å)	0.0304 ± 0.0015	0.0310
Dihedral (°)	1.6718 ± 0.1814	1.7120
From idealized covalent geometry		
Bonds (Å)	0.0047 ± 0.0002	0.0045
Angles (°)	0.8323 ± 0.0130	0.8400
Impropers (°)	0.9045 ± 0.0231	0.9020
Ramachandran plot statistics		
% of residues in most favorable region of Ramachandran plot	84.4	85.7
% of residues in disallowed region of Ramachandran plot	0.0	0.0

C. Superimposition on the representative structure (Å)

Backbone (C, Cα, N) RMSD over the structured protein core (residues 10-207, 226-239)	0.88 ± 0.07

[a] <S> is the ensemble of 20 final structures; S_{rep} is the representative structure, selected from the final family on the criteria of having the lowest sum of pairwise RMSD for the remaining structures in the family.

Supplementary Table 2. Statistics for the ensemble of the calculated 20 structures of the apo-form of WBSCR27.

A. Restraints used in the structure calculation

Total distance restraints	2355	Total dihedral angles	295
Long range (|i-j| > 4)	430	Phi (ϕ)	148
Medium (1 < |i-j| < 5)	264	Psi (ψ)	147
Sequential (|i-j| = 1)	668		
Intraresidue	881	RDCs measured in Pf1	143
Hydrogen bonds	112		

B. Restraint violations and structural statistics (for 20 structures)

No NOE or dihedral angle violations are above 0.5 Å and 5° respectively.

Average RMSD	<S>[a]	S_{rep}
From experimental restraints		
Distance (Å)	0.0268 ± 0.0008	0.0260
Dihedral (°)	1.1901 ± 0.0901	1.1840
From idealized covalent geometry		
Bonds (Å)	0.0036 ± 0.0002	0.0035
Angles (°)	0.5773 ± 0.0122	0.5780
Impropers (°)	0.6023 ± 0.0168	0.6050
Ramachandran plot statistics		
% of residues in most favorable region of Ramachandran plot	87.2	87.2
% of residues in disallowed region of Ramachandran plot	0.0	0.0

C. Superimposition on the representative structure (Å)

Backbone (C, Cα, N) RMSD over the structured protein core (residues 52-206, 228-238)	1.07 ± 0.15

[a] <S> is the ensemble of 20 final structures; S_{rep} is the representative structure, selected from the final family on the criteria of having the lowest sum of pairwise RMSD for the remaining structures in the family.

Supplementary Table 3. Biotinylated proteins identified by panoramic proteome analysis in the BioID experiment. The cell lines expressing HA-BirA* and the ones expressing HA-BirA*-WBSCR27 were studied in 3 replicas. Data shown in Excel format (SupplementaryTable8.xlsx). List 1. Names of the identified proteins in all experiments. Lists 2-7. The results of protein identification in each experiment.

Supplementary Table 4. Oligonucleotides used for the construction of fusions HA-WBSCR27 and mKate2-WBSCR27.

Primer name	Sequence (5' - 3')
WB27-pSBtet-fwd	AAAAAAAGGCCTCTGAGGCCATGGCTCAGGAGGAGGCTGG
WB27-pSBtet-rev	AAAAAAAGGCCTGACAGGCCTCATACTGTCTCCTGCTTCCGGTAAAGGT
WB27-pSBtet-HA-fwd	GAACATCATATGGATATACTGTCTCCTGCTTCCGGTAAAGG
WB27-pSBtet-HA-rev	CAGATTATGCTTAGGGTGAGAGAGGGAGGTTCAGTATC
WB27-pSBtet-mKate2-fwd	CCTCGAAAGGCCTCTGAGGCCAATGAGCGAGCTGATTAAGGAG
WB27-pSBtet-mKate2-rev	CGCCCAGCCTCCTCCTGAGCCAGGCGTGCCCCAGTTTGCT

Supplementary Table 5. Oligonucleotides used for endogenous C-terminal HA-tagging of WBSCR27 into NIH3T3 cells.

Primer name	Sequence (5' - 3')
WB-Cterm1-fwd	CACCGCCGGAAGCAGGAGACAGTAT
WB-Cterm1-rev	AAACATACTGTCTCCTGCTTCCGGC
WB-Cterm2-fwd	CACCGCGGAAGCAGGAGACAGTATA
WB-Cterm2-rev	AAACTATACTGTCTCCTGCTTCCGC
Human_U6_Seq_Fw_Insert	ACTATCATATGCTTACCGTAAC
WB27Cterm_check_F	ACCAACCCATCCAACCTTCC
WB27Cterm_check_R	GAGCCCTGGGTCCAAAGAAA
HA-ins-fwd	CAGATTATGCTTAGGGTGAGAGAGGGAGGTTCAGTATC
HA-ins_rev	GAACATCATATGGATATACTGTCTCCTGCTTCCGGTAAAGG
pUC_Fw	GTAAAACGACGGCCAGT
pUC_Rev	CAGGAAACAGCTATGAC
WB27_C-term_HA_ins_check_Rev	TCGGCTACCTGCTGAGTGACC
WB27_C-term_HA_ins_check_Rev	GATGTCTCAATAGCGCCTCCTG

Supplementary Table 6. Primers used to generate and validate WBSCR27 knockouts.

Primer name	Sequence (5' - 3')
WBSCR27_3_F	CACCGCCCCCGCAGGAGAATGACTC
WBSCR27_3_R	AAACGAGTCATTCTCCTGCGGGGGC
Human_U6_Seq_Fw_Insert	ACTATCATATGCTTACCGTAAC
WBSCR27_RT_F	GCACTGATAACCTCCTCCGCCGGG
WBSCR27_RT_R	CCGGAGCCCAGTCGTCATAGAAGC

Supplementary Table 7. Primers used for WBSCR27-BirA generation.

Primer name	Sequence (5' - 3')
After_eGFP_SfiI_ins_Fw	GGCCTGTCAGGCCGGCGCGCCACTTCTAAATAAG
BirA_C-term_Rev	CTTCTCTGCGCTTCTCAGGGA
ADH1_terminator_seq	CCGGTAGAGGTGTGGTCAAT
BirA_N-HA_ins_Fw	ATGTACCCATACGATGTTCCAGATTACGCTATGGACAAGGACAACACCGT
BirA_N-HA_SfiI_ins_Rev	GGCCTCAGAGGCCTTTAATTAAACCAGCACCGTCACCG
HA-tag_Fw	TACCCATACGATGTTCCAGATTACGCT
BirA_N-HA_SfiI_ins_check_Rev	TGCCAGCCTGCTGGTACTC
N-HA_BirA-WBSCR27_Fw	CTCCCTGAGAAGCGCAGAGAAGGCCATGGCTCAGGAGG
N-HA_BirA-WBSCR27_Rev	GCGCGCCGGCCTGACAGGCCTCATACTGTCTCCTGCTTCCG
N-HA_BirA-WBSCR27_check_Fw	AGGAGGGACTGGCTCCTTAC
N-HA_BirA-WBSCR27_check_Rev	TGCTTCAGCATTTCTGGGCTT
pSBbi-GN_WBSCR27_BirA_N-HA_seq_Fw_1	AACAGATGGCTGGCAACTAGA
pSBbi-GN_WBSCR27_BirA_N-HA_seq_Fw_2	TGTCTTGCCTGTCAGCTCC
pSBbi-GN_WBSCR27_BirA_N-HA_seq_Fw_3	GCTCGCCAGAGTGGAACT
pSBbi-GN_WBSCR27_BirA_N-HA_seq_Fw_4	TGCAGCTCCACAGCTACCAG
BirA_fwd	TgAggCCTgTCAggCCAAg
BirA_rev	CTTCTCTgCgCTTCTCAgggAg

Supplementary Table 8. Chemical shifts of SAH in free and bound to WBSCR27 states.

Position	Free SAH		Bound SAH	
	^{1}H, ppm	^{13}C, ppm	^{1}H, ppm	^{13}C, ppm
2	8.28	155.8	8.80	156.9
8	8.36	142.9	7.69	138.5
1’	6.09	90.2	6.08	87.1
2’	4.87	76.0	4.18	76.2
3’	4.43	75.0	4.06	71.4
4’	4.34	86.2	4.02	83.9
5’	2.99, 3.07	36.3	2.78, 2.91	37.7
α	3.80	56.5	3.81	56.7
β	2.67, 2.72	30.6	2.57, 2.95	31.0
γ	2.07, 2.15	33.2	2.02, 2.16	31.8

2.2 Supplementary Figures

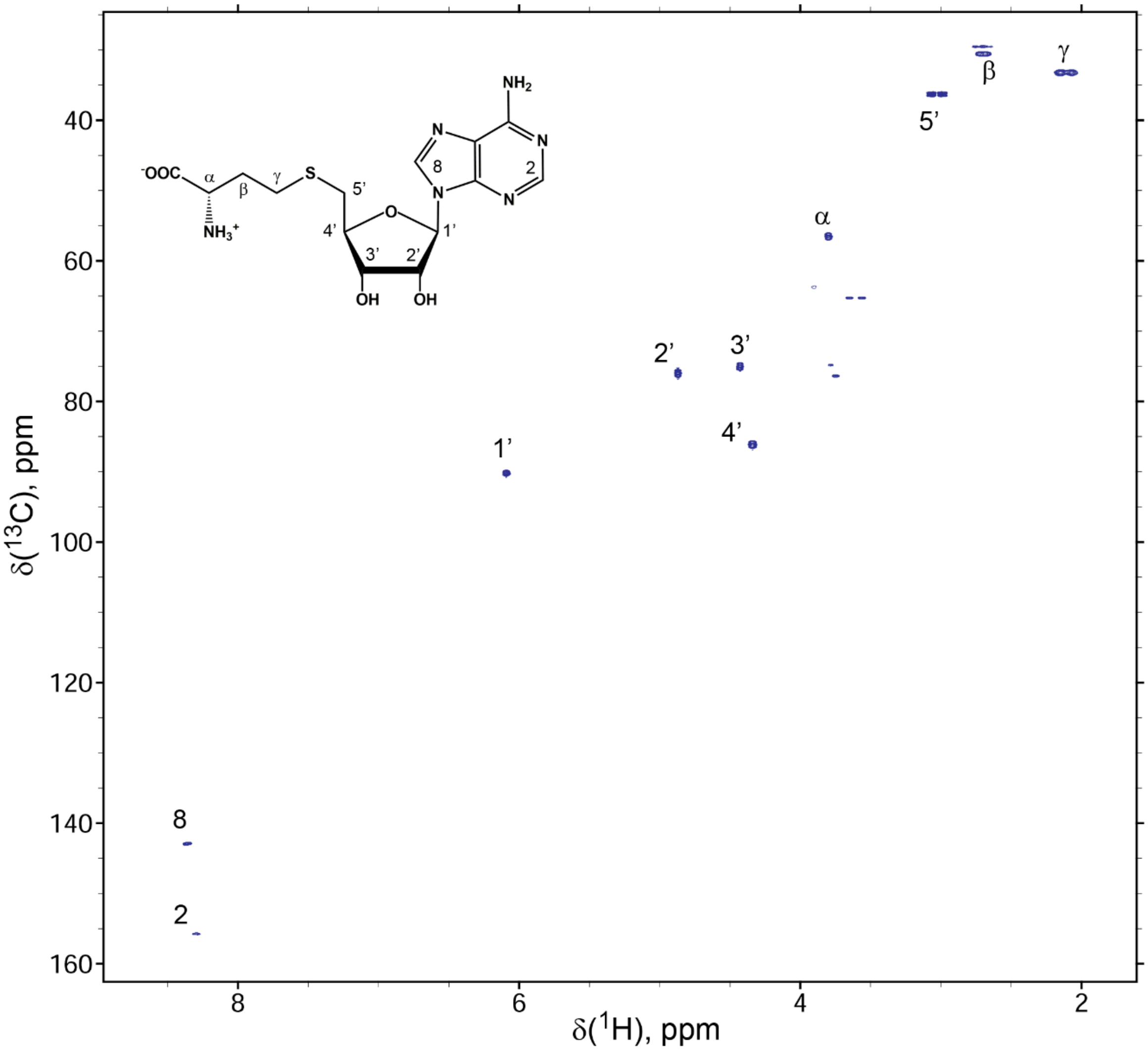

Supplementary Figure S1. 2D ^{1}H-^{13}C HSQC spectrum of ^{13}C-labelled SAH isolated from *E. coli* BL21 (DE3 pLysS) cell culture grown on M9 minimal medium containing ^{13}C and ^{15}N isotopes. Shown are resonance assignments. Spectrum was recorded in D_2O, in presence of 50 mM phosphate buffer (pH 7.0), at 35°C and 600 MHz proton resonance frequency.

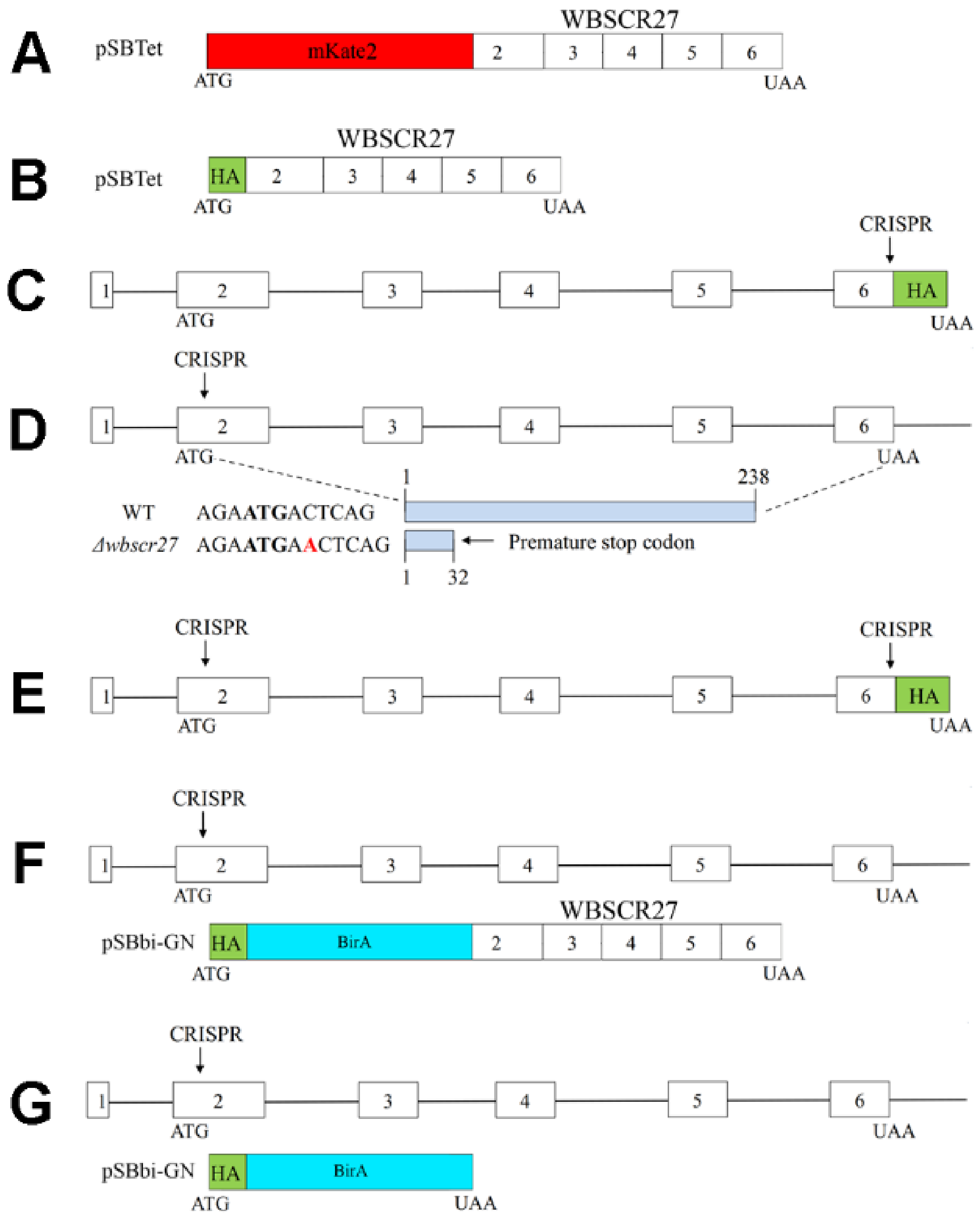

Supplementary Figure S2. NIH3T3 based cell lines created in this study. **A**. Cells with ectopic expression of the N-terminal fusion mKate2-WBSCR27 used to study protein localization within the cells. **B**. Cells with ectopic expression of the N-terminal fusion HA-WBSCR27 used to study protein localization, as well as for co-immunoprecipitation experiments. **C**. Cell line with endogenous C-terminal WBSCR27-HA fusion used in co-immunoprecipitation experiments. **D**. Knockout line containing biallelic point mutation in 2nd exon used to study the phenotypic consequences of WBSCR27 depletion. The premature stop codon appearing due to the frame-shift results in a 32 amino acid product (shown below the diagram). **E**. The knockout cells with C-terminal HA-tag used to check the WBSCR27 depletion on a protein level. **F**, **G**. Two cell lines based on knockout line with ectopic expression of HA-BirA*-WBSCR27 and HA-BirA* respectively, constructed for proximal labelling in BioID experiment.

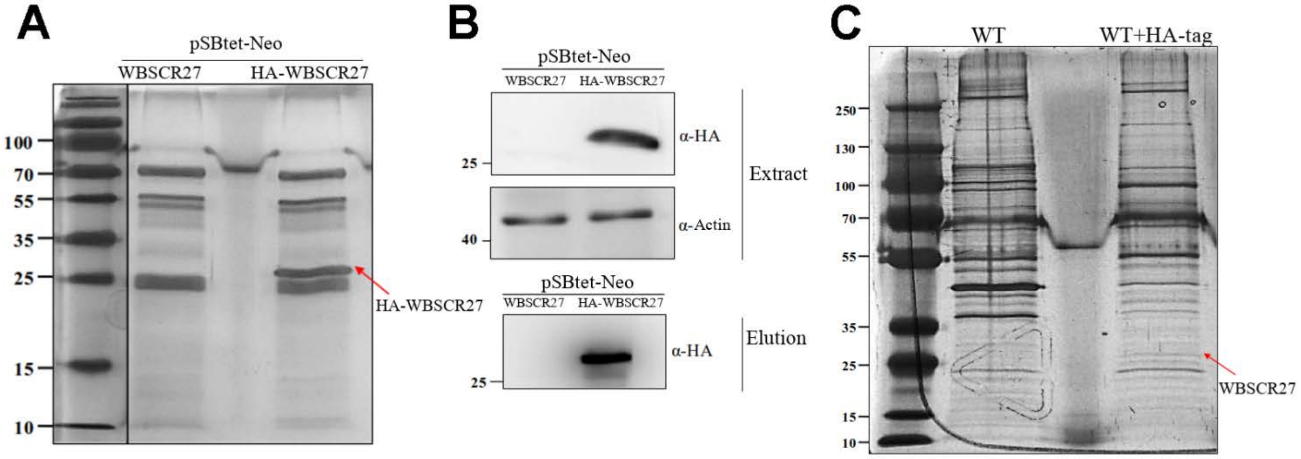

Supplementary Figure S3. Lack of partner proteins co-immunoprecipitated with HA-WBSCR27. Silver stained gels (A, C) and anti-HA immunoblots (B) with the eluates from anti-HA resin for the control NIH3T3 cells and NIH3T3 cells with ectopic expression of HA-WBSCR27 (A, B) and expression of WBSCR27-HA from natural *Wbscr27* promoter (C).

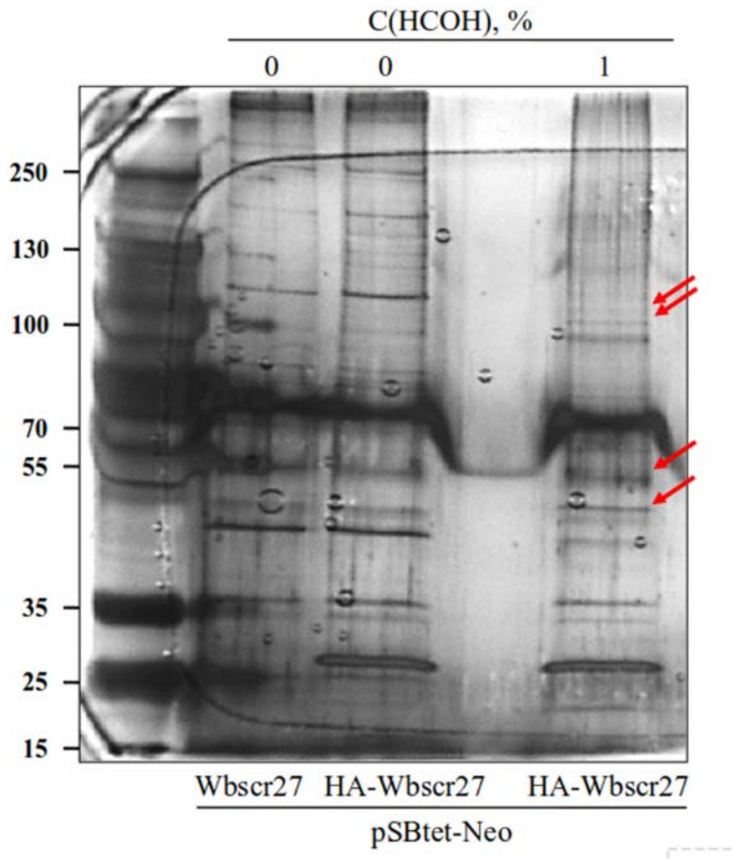

Supplementary Figure S4. Lack of partner proteins co-immunoprecipitated with HA-WBSCR27 following formaldehyde cross-linking. Silver stained gel with the eluates from anti-HA resin for the control NIH3T3 cells with ectopic expression of HA-WBSCR27 correspondingly. Arrows points to lines potentially distinguishing knockout from control.

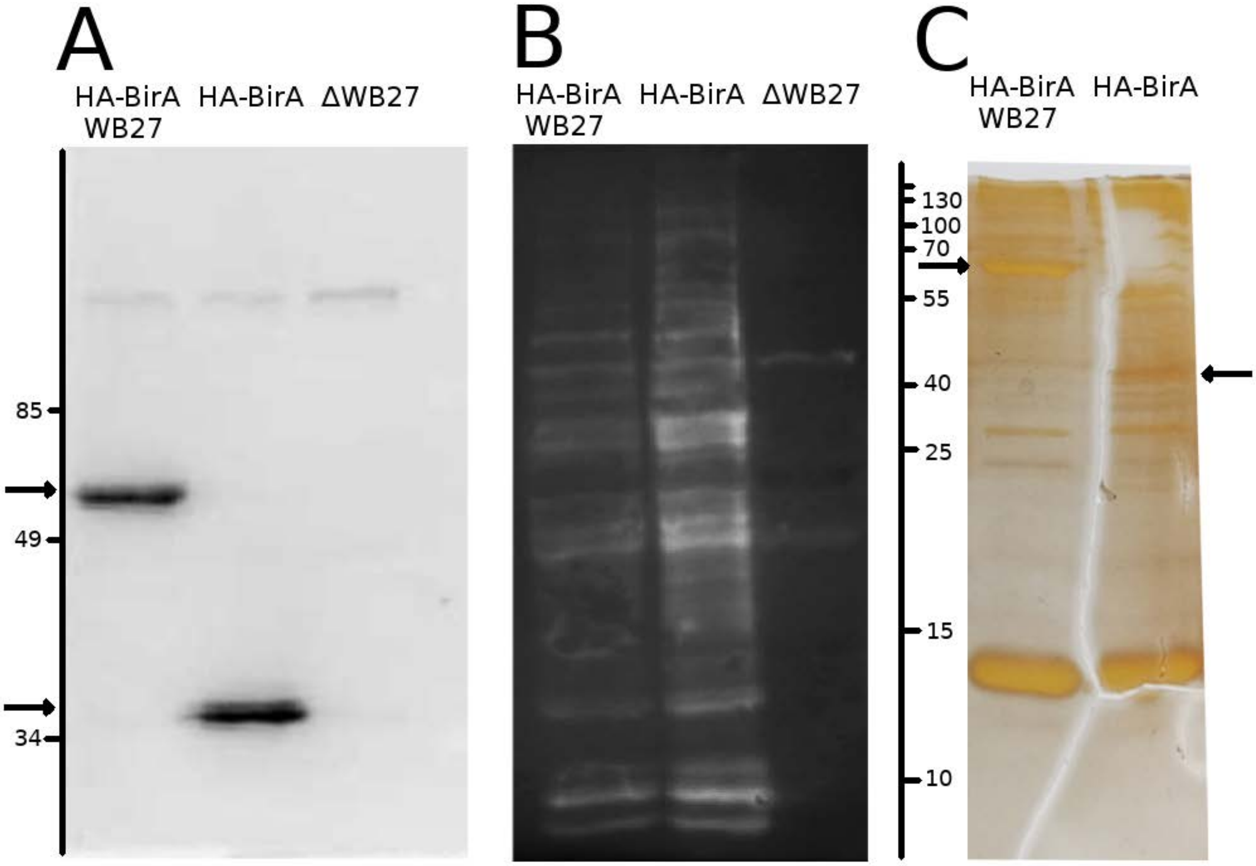

Supplementary Figure S5. Lack of partner proteins of WBSCR27 identified with BioID. (A) Expression of HA-BirA*-WBSCR27 (left lane), HA-BirA* (central lane) constructs and parental NIH3T3 cell line visualized using anti-HA antibody. (B) Staining of the biotinylated proteins in the cell lines expressing HA-BirA*-WBSCR27 (left lane), HA-BirA* (central lane) and parental NIH3T3 cell line (right lane) using streptavidin-HRP. (C) Silver stained gel with the eluates from streptavidin resin for the cells expressing HA-BirA*-WBSCR27 (left lane) and HA-BirA* (right lane). The arrows point to HA-BirA*-WBSCR27 and HA-BirA* lines.

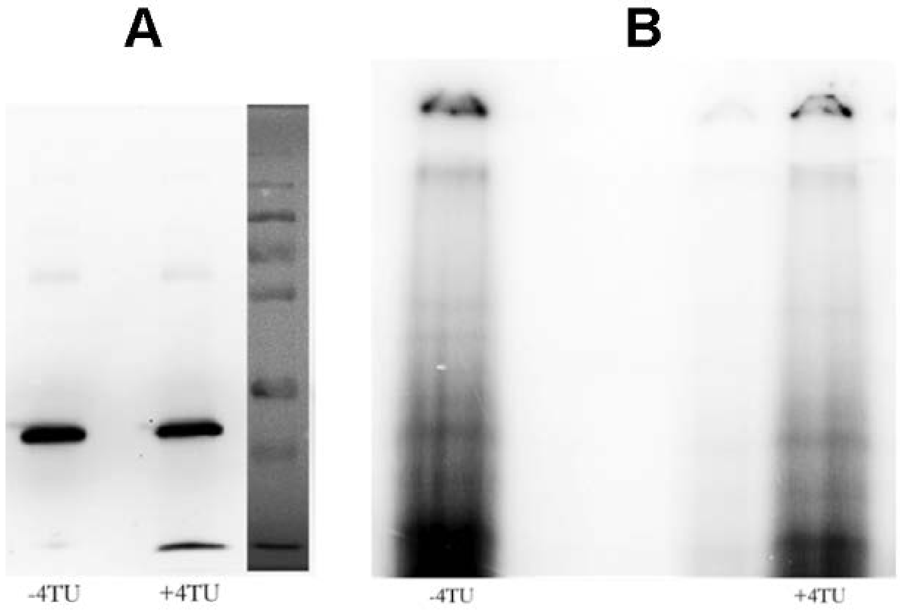

Supplementary Figure S6. PAR-CLIP analysis of WBSCR27 RNA partners. **A**. Anti-HA immunoblotting of HA-WBSCR27 following immunopurification of the cross-linked complexes. **B**. Cross-linked RNA visualization by autoradiography. No enrichment of RNA cross-linked to HA-WBSCR27 is evident depending on the 4-thiouridine presence.

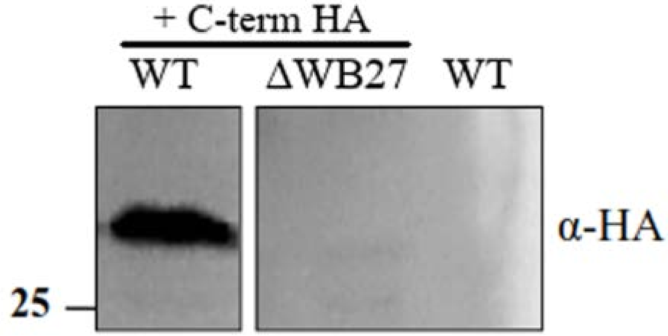

Supplementary Figure S7. Inactivation of the WBSCR27 gene in the NIH3T3 cell line carrying biallelic WBSCR27-HA gene variants. Shown are the results of immunoblotting of cell lysates with anti-HA antibodies.

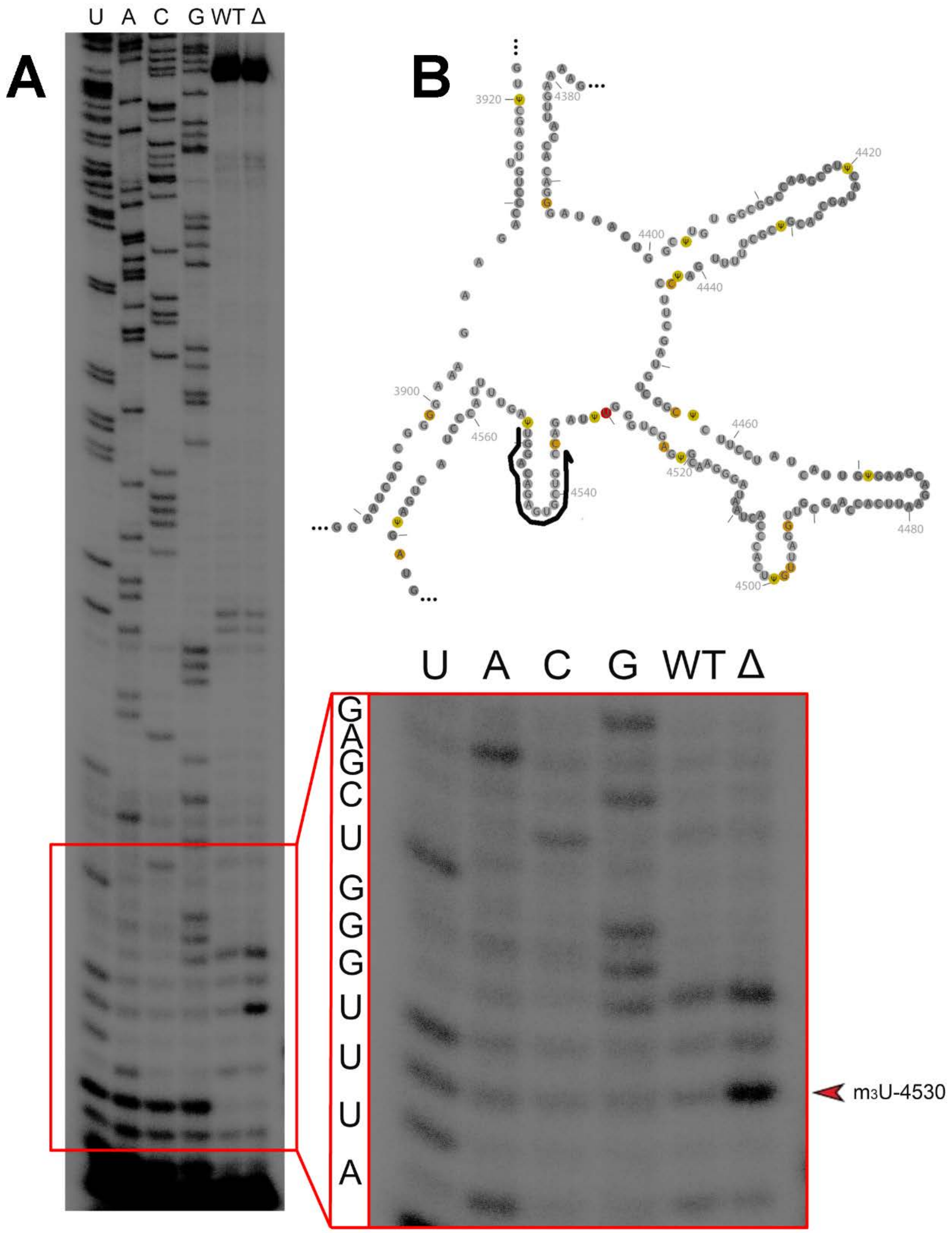

Supplementary Figure S8. The primer extension assay for probing the U4530 methylation of the 28S rRNA. **A**. Results of electrophoresis in the 10% (w/v) denaturing polyacrylamide gel. U, A, C, and G correspond to the sequencing lanes. Primer extension products in the WT lane corresponds to the RNA extracted from the wild-type NIH3T3 cells; Δ refers to the RNA extracted from the WBSCR27 knockout NIH3T3 strain. The primer used in experiment was complementary to the fragment 4537–4551 of the 28S rRNA. **B**. Schematic representation of the secondary structure of the 28S rRNA fragment. Marked in red is m^3U4530. All other methylated nucleotides are highlighted in orange. Pseudouridine nucleotides are highlighted in yellow. The arrow shows the reverse primer.

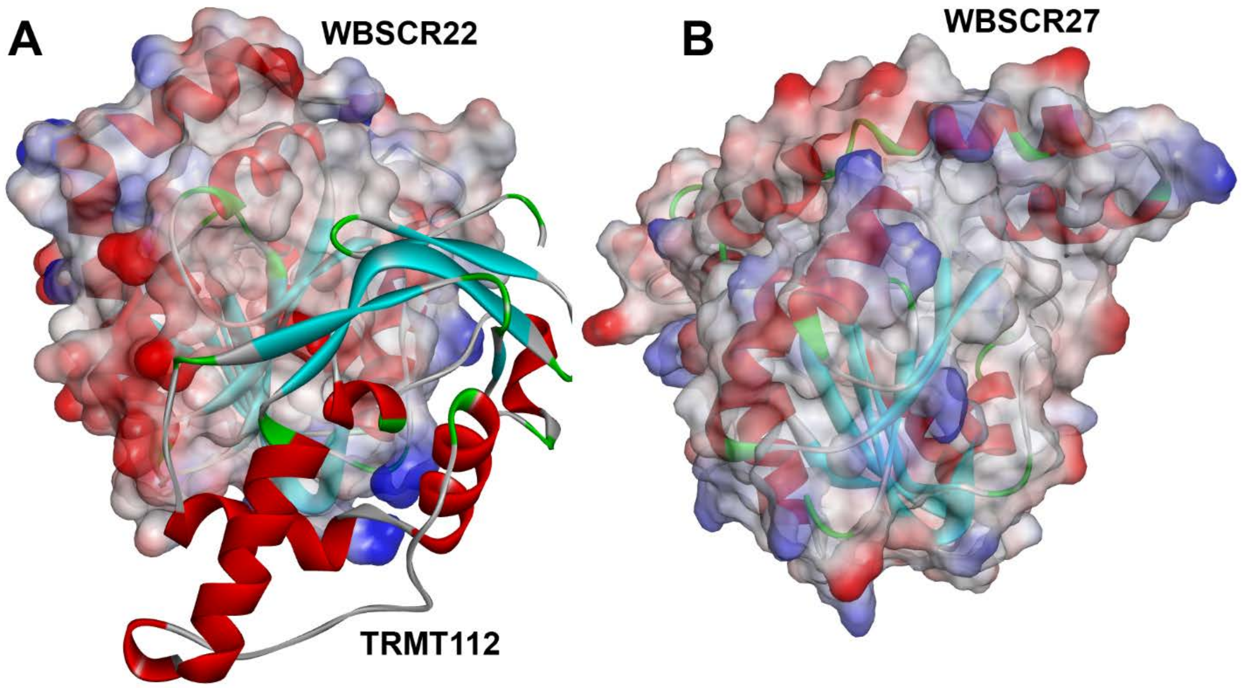

Supplementary Figure S9. Solvent accessible molecular surfaces of (**A**) WBSCR22 (Bud23) in a complex with TRMT112 (PDB id 4QTU) and (**B**) WBSCR27 in a complex with SAH (PDB id 7QCB). Molecular surfaces are colored according to electrostatic surface potential.

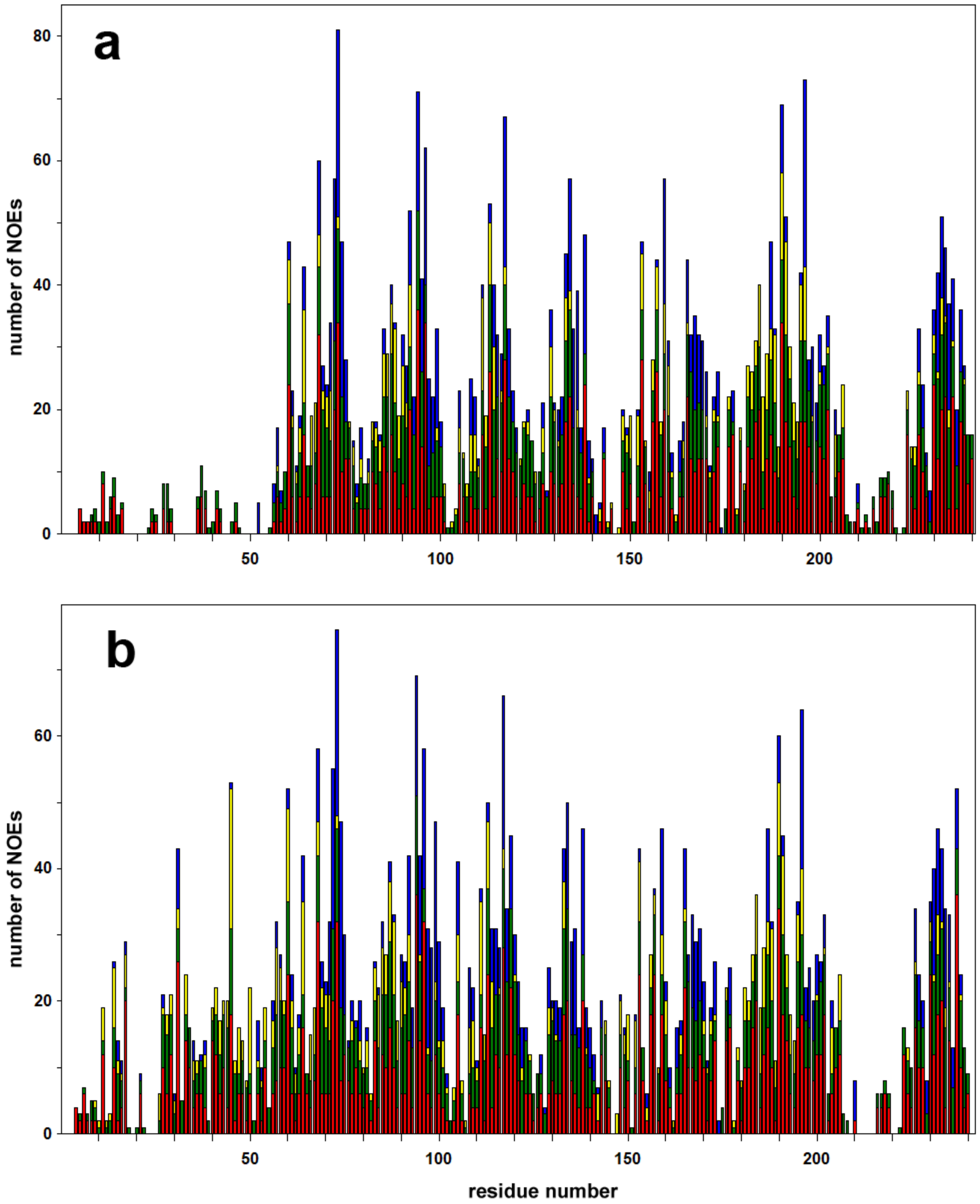

Supplementary Figure S10. NOE histograms giving the number of long-range (blue), medium-range (yellow), sequential (green) and intra-residue NOEs for each protein residue in structure calculation of the apo-form of WBSCR27 (**a**) and WBSCR27-SAH complex (**b**).

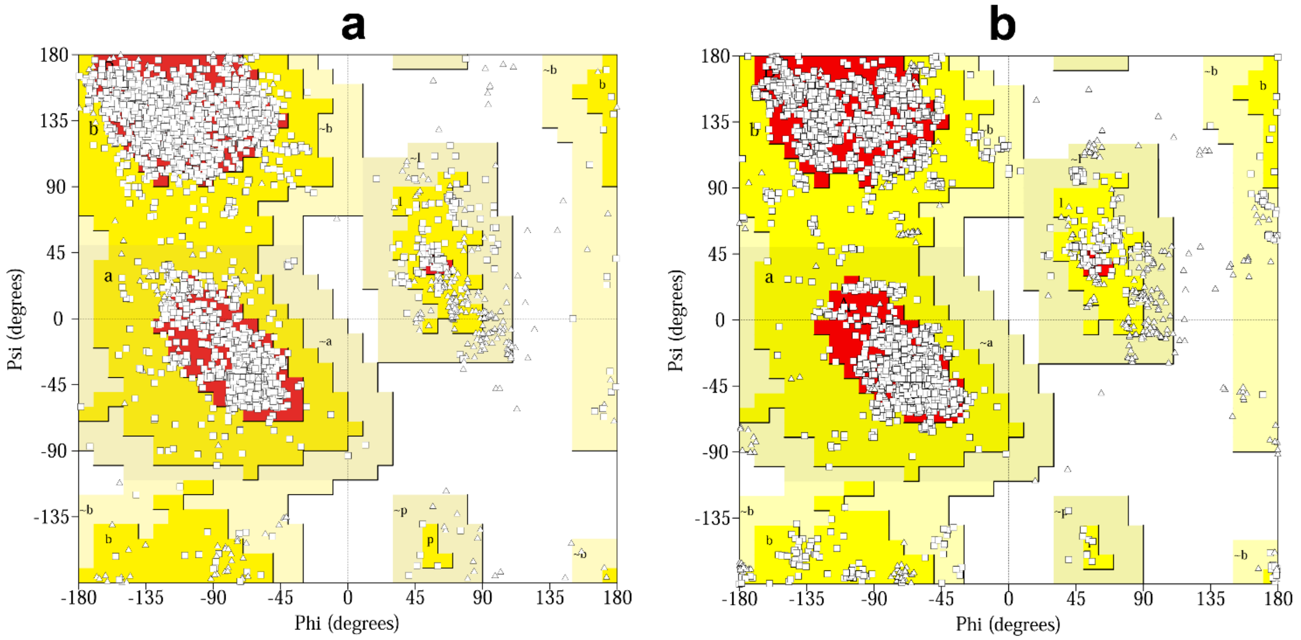

Supplementary Figure S11. The Ramachandran plots for the final 20 structures of the apo-form of WBSCR27 (**a**) and WBSCR27-SAH complex (**b**). No residues fall in disallowed regions. 87.2 % and 84.4 % of residues fall in the most favorable regions in apo-form and WBSCR27-SAH complex.

3 Supplementary References

Bai, Y., Milne, J.S., Mayne, L., and Englander, S.W. (1993). Primary structure effects on peptide group hydrogen exchange. *Proteins: Structure, Function, and Bioinformatics* 17(1)**,** 75-86. doi: 10.1002/prot.340170110.

Brunger, A.T., Adams, P.D., Clore, G.M., DeLano, W.L., Gros, P., Grosse-Kunstleve, R.W., Jiang, J.-S., Kuszewski, J., Nilges, M., Pannu, N.S., Read, R.J., Rice, L.M., Simonson, T., and Warren, G.L. (1998). Crystallography & NMR System: A New Software Suite for Macromolecular Structure Determination. *Acta Crystallogr D* 54(5)**,** 905-921. doi: doi:10.1107/S0907444998003254.

Delaglio, F., Grzesiek, S., Vuister, G.W., Zhu, G., Pfeifer, J., and Bax, A. (1995). NMRPipe: a multidimensional spectral processing system based on UNIX pipes. *J Biomol NMR* 6(3)**,** 277-293.

Farrow, N.A., Muhandiram, R., Singer, A.U., Pascal, S.M., Kay, C.M., Gish, G., Shoelson, S.E., Pawson, T., Forman-Kay, J.D., and Kay, L.E. (1994). Backbone dynamics of a free and phosphopeptide-complexed Src homology 2 domain studied by ^{15}N NMR relaxation. *Biochemistry* 33(19)**,** 5984-6003.

Kowarz, E., Löscher, D., and Marschalek, R. (2015). Optimized Sleeping Beauty transposons rapidly generate stable transgenic cell lines. *Biotechnology Journal* 10(4)**,** 647-653. doi: https://doi.org/10.1002/biot.201400821.

Kuszewski, J., Gronenborn, A.M., and Clore, G.M. (1997). Improvements and extensions in the conformational database potential for the refinement of NMR and X-ray structures of proteins and nucleic acids. *J Magn Reson* 125(1)**,** 171-177. doi: 10.1006/jmre.1997.1116.

Laskowski, R.A., MacArthur, M.W., Moss, D.S., and Thornton, J.M. (1993). PROCHECK: a program to check the stereochemical quality of protein structures. *Journal of Applied Crystallography* 26(2)**,** 283-291. doi: doi:10.1107/S0021889892009944.

Mátés, L., Chuah, M.K.L., Belay, E., Jerchow, B., Manoj, N., Acosta-Sanchez, A., Grzela, D.P., Schmitt, A., Becker, K., Matrai, J., Ma, L., Samara-Kuko, E., Gysemans, C., Pryputniewicz, D., Miskey, C., Fletcher, B., VandenDriessche, T., Ivics, Z., and Izsvák, Z. (2009). Molecular evolution of a novel hyperactive Sleeping Beauty transposase enables robust stable gene transfer in vertebrates. *Nat Genet* 41(6)**,** 753-761. doi: 10.1038/ng.343.

Molday, R., Englander, S., and Kallen, R. (1972). Primary structure effects on peptide group hydrogen exchange. *Biochemistry* 11(2)**,** 150-158. doi: 10.1021/bi00752a003.

Polshakov, V.I., Morgan, W.D., Birdsall, B., and Feeney, J. (1999). Validation of a new restraint docking method for solution structure determinations of protein–ligand complexes. *Journal of Biomolecular NMR* 14(2)**,** 115-122. doi: 10.1023/a:1008379225053.

Polshakov, V.I., Birdsall, B., Frenkiel, T.A., Gargaro, A.R., and Feeney, J. (1999). Structure and dynamics in solution of the complex of *Lactobacillus casei* dihydrofolate reductase with the new lipophilic antifolate drug trimetrexate. *Protein Sci* 8(3)**,** 467-481. doi: 10.1110/ps.8.3.467.

Ran, F.A., Hsu, P.D., Wright, J., Agarwala, V., Scott, D.A., and Zhang, F. (2013). Genome engineering using the CRISPR-Cas9 system. *Nature Protocols* 8(11)**,** 2281-2308. doi: 10.1038/nprot.2013.143.

Tjandra, N., and Bax, A. (1997). Direct measurement of distances and angles in biomolecules by NMR in a dilute liquid crystalline medium. *Science* 278(5340)**,** 1111-1114. doi: 10.1126/science.278.5340.1111.

Trempe, J.-F., Morin, F.G., Xia, Z., Marchessault, R.H., and Gehring, K. (2002). Characterization of polyacrylamide-stabilized Pf1 phage liquid crystals for protein NMR spectroscopy. *Journal of Biomolecular NMR* 22(1)**,** 83-87. doi: 10.1023/a:1013832422428.
